# IL-6/STAT3 signaling in tumor cells restricts the expression of frameshift-derived neoantigens by SMG1 induction

**DOI:** 10.1186/s12943-022-01679-6

**Published:** 2022-11-28

**Authors:** Daniel Meraviglia-Crivelli, Helena Villanueva, Angelina Zheleva, María Villalba-Esparza, Beatriz Moreno, Ashwathi Puravankara Menon, Alfonso Calvo, Javier Cebollero, Martin Barainka, Igor Ruiz de los Mozos, Carlos Huesa-Berral, Fernando Pastor

**Affiliations:** 1 Molecular Therapeutics Program, Center for Applied Medical Research, CIMA, University of Navarra, 31008 Pamplona, Spain; 2 Instituto de Investigación Sanitaria de Navarra (IDISNA), Recinto de Complejo Hospitalario de Navarra, 31008 Pamplona, Spain; 3 Department of Pathology, Yale University School of Medicine, New Haven, CT 06510 USA; 4 IDISNA, CIBERONC, Program in Solid Tumors (CIMA), Department of Pathology, Anatomy and Physiology, School of Medicine, University of Navarra, Avenida Pío XII, 55, 31008 Pamplona, Spain; 5 Gene Therapy Program, Center for Applied Medical Research, CIMA, University of Navarra, 31008 Pamplona, Spain; 6 Department of Personalized Medicine, NASERTIC, Government of Navarra, 31008 Pamplona, Spain; 7 Department of Physics and Applied Mathematics, School of Science, University of Navarra, E-31008 Pamplona, Navarra Spain; 8 Department of Molecular Therapies, CIMA (Center for Applied Medical Research) University of Navarre, Av. de Pío XII, 55, 31008 Pamplona, Spain

**Keywords:** Tumor immunity, Neoantigens, Cancer immunotherapy, Immunoediting, NMD

## Abstract

**Background:**

The quality and quantity of tumor neoantigens derived from tumor mutations determines the fate of the immune response in cancer. Frameshift mutations elicit better tumor neoantigens, especially when they are not targeted by nonsense-mediated mRNA decay (NMD). For tumor progression, malignant cells need to counteract the immune response including the silencing of immunodominant neoantigens (antigen immunoediting) and promoting an immunosuppressive tumor microenvironment. Although NMD inhibition has been reported to induce tumor immunity and increase the expression of cryptic neoantigens, the possibility that NMD activity could be modulated by immune forces operating in the tumor microenvironment as a new immunoediting mechanism has not been addressed.

**Methods:**

We study the effect of SMG1 expression (main kinase that initiates NMD) in the survival and the nature of the tumor immune infiltration using TCGA RNAseq and scRNAseq datasets of breast, lung and pancreatic cancer. Different murine tumor models were used to corroborate the antitumor immune dependencies of NMD. We evaluate whether changes of SMG1 expression in malignant cells impact the immune response elicited by cancer immunotherapy. To determine how NMD fluctuates in malignant cells we generated a luciferase reporter system to track NMD activity in vivo under different immune conditions. Cytokine screening, in silico studies and functional assays were conducted to determine the regulation of SMG1 via IL-6/STAT3 signaling.

**Results:**

IL-6/STAT3 signaling induces SMG1, which limits the expression of potent frameshift neoantigens that are under NMD control compromising the outcome of the immune response.

**Conclusion:**

We revealed a new neoantigen immunoediting mechanism regulated by immune forces (IL-6/STAT3 signaling) responsible for silencing otherwise potent frameshift mutation-derived neoantigens.

**Supplementary Information:**

The online version contains supplementary material available at 10.1186/s12943-022-01679-6.

## Introduction

Cancer immunotherapy has changed the clinical outcome in a subset of cancer patients due to the success of immune-checkpoint blockade (ICB) antibodies and adoptive cell therapies [1, 2]. Despite the promising horizon of cancer immunotherapy, many cancer patients do not show any clinical benefits from immunotherapy yet. There are dozens of clinical trials combining the US *Food and Drug Administration* (FDA) approved ICB, among others and with new therapeutic targets in a quest to broaden the range of patient response to immunotherapy [3]. Currently the most successful FDA approved combination includes concomitant blockade of anti-PD-1 and anti-CTLA-4 [3].

A possible explanation for the insufficient treatment responses in many cancer patients is a lack of tumor antigenicity due to a limited mutational load, and pervasiveness of tumor antigen immunoediting. Tumor antigenicity is indeed an important factor determining the effectiveness of an immune response conditioned by its intrinsic mutation load [4] and the selective immune pressure (antigen immunoediting) of each tumor lesion [5]. Although tools exist to reinvigorate a preexisting antitumor immune response (e.g., ICB), it is more challenging to develop therapeutic interventions that improve the quality of basal antigen landscape of each tumor. Tumor antigenicity is also probably affected by the quality of mutations accumulated along tumor ontogeny. Not all mutations are equally able to induce potent neoantigens [6]. Single-point mutations will, in the best-case scenario, elicit change in only one amino acid of the peptide sequence. Nonetheless, frameshift mutations derived from “indels” (insertion or deletion) or translocations can trigger a shift in the open reading frame of the protein leading to the translation of *de novo* protein with an alternative (“foreign”) amino acid sequence, whereby a broader range of neoepitope peptides can be efficiently presented and recognized by the immune system as a potential foreign antigen [6]. The occurrence of indel mutations, unfortunately, is underestimated with conventional methods of next-generation sequencing [7–9]. Moreover, they are often eliminated by NMD, as they usually contain premature stop codons (PTCs) [10]. These factors (mutation load, indel frequencies and NMD dependencies) may be key in determining the expanse of the overall tumor neoantigen-landscape [4, 6, 11, 12].

NMD is a conserved RNA surveillance mechanism involved in the elimination of mRNA with premature termination codons (PTCs) derived from pre-mRNA maturation errors or from DNA mutations. During splicing, the exon junction complex (EJC) remained bound to mRNA until it is displaced by the ribosome during the first round of translation. The persistence of EJC upstream of a PTC lead to the initiation of NMD cascade, recruiting NMD factors compose of UPF1, UPF2, UPF3B, SMG1, etc. [13]. The first factor that license the action of NMD by phosphorylation of UPF1 is the SMG1 kinase [13].

Considering the potential role of NMD in the antigen landscape, as its inhibition triggers tumor immunity by stabilization of PTC-containing neoantigens [14–17], we hypothesize that NMD function could be actively sequestered by the tumor to silence frameshift-derived neoantigens.

We identify the existence of a molecular mechanism shielding the tumor from immune attack regulated by the IL-6/STAT3/SMG1 axis that prevents antitumor immune responses: a new immunoediting process to silence potent neoantigens by immune intrinsic pathways that affect the activity of NMD. IL-6/ IL-6R blockade has shown very promising results in preclinical models [18–20] and is evaluated in clinical trials in combination with ICB; (breast cancer: ClinicalTrials.gov NCT03424005; Pancreatic Cancer: NCT04258150; Non-Small Cell Lung Cancer: NCT04940299, NCT04691817; Melanoma: NCT03999749). With this current study, we shed light on a new immunosuppressive mechanism of action of the IL-6/STAT3 axis modulating the expression of potent neoantigens under the control of NMD.

## Results

### High SMG1 expression is correlated to worse survival and is associated with lower immune infiltration in breast cancer (BRCA), Lung Adenocarcinoma (LUAD) and Pancreatic Adenocarcinoma (PAAD) patients

**Fig. 1:**
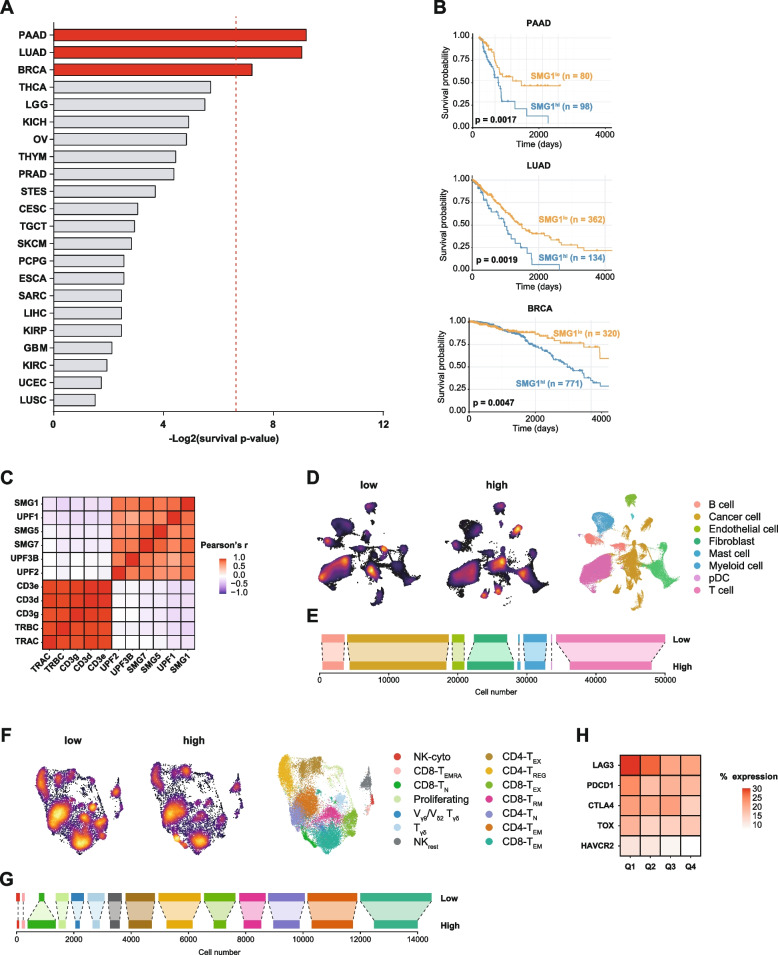
High SMG1 expression correlates with worse survival and lower immune infiltration in some tumors. (**A**) Patients with different tumors from TCGA cohorts were classified in two categories according to their SMG1 expression levels: ‘low’ or ‘high’ and significance of long-term survival was determined using long-rank test. Statistical significance was set in p ≤ 0.01 (dot line). n ≥ 100 patients per cohort. (**B**) Kaplan-Meyer curves of statistically significant tumors from (A): PAAD, LUAD and BRCA. (**C**) Heatmap illustrating correlations between the expression of genes for NMD factors and immune response-related markers in BRCA patients. scRNAseq expression data reanalyzed [21]. Heatmap shows Pearson’s r coefficient. (**D**) Uniform Manifold Approximation and Projection (UMAP) map of breast cancer patient [21] classified depending on their SMG1 expression as ‘high’ or ‘low’ based on the SMG1 expression on tumor cells from pretreatment samples (*n*= 31). Each cell type is determined by color code. (**E**) Bar plot showing absolute cell numbers of each population shown in (D). (**F**) Differentiated T-cell subpopulations in BRCA patients with low and high SMG1 expression levels as in (D). UMAP clusters of 14 different cell types depicted by color code T cells were classified as naive (CD4+ TN and CD8+ TN), regulatory T cells (CD4+ TREG), effector/memory T cells (CD4+ TEM and CD8+ TEM), recently activated effector/memory T cells (CD8+TEMRA), tissue-resident memory T cells (CD8+ TRM), exhausted T cells (CD4+ and CD8+ TEX) and proliferating T cells, resting NK cells (NKres) and cytotoxic NK cells (NKcyto), gamma-delta T cells with semi-invariant T-cell repertoires (Vγ9/Vδ2 Tγδ) and with memory features (Tγδ). (**G**) Bar plot showing absolute cell numbers of each population shown in (F). (**H**) Heatmap showing the abundance exhaustion markers on T cells in samples with different SMG1 expression as in (D), but SMG1 expression was divided in 4 quartiles from low to high: Q1, Q2, Q3 and Q4. Percentage of positive cells for each of the main exhaustion markers is shown (LAG3, PDCD1, CTLA4, TOX and HAVCR2)

To evaluate the role of SMG1 as the main kinase that controls the activation of NMD in different human cancers, we interrogated the TCGA dataset encompassing all cancer types with their reported survival data. 22 different cancer entities were analyzed for their survival according to the expression levels of SMG1. In three prevalent tumor types (BRCA, LUAD and PAAD), high expression of SMG1 was significantly associated with worse prognosis of patients (Fig. 1). In particular, the stratification of cancer patients based on tumor SMG1 expression led to survival predicted *p*-values of 0.0017 for PAAD, 0.0019 for LUAD and 0.0047 for BRCA (Fig. 1B).

To ascertain whether this predicted overall survival rates were related to a stronger antitumor immune responses in patients with lower levels of NMD, we analyzed three independent scRNAseq dataset collected from 31 BRCA patients [21], from 11 LUAD patients [22], and from 24 PDAC patients (the most common type of PAAD) [23]. For each tumor type, the levels of the key NMD factors were positively associated between all tumor cells, and inversely correlated with expression of T-cell markers (CD3, TCR) (Fig. 1C, S1A, S1D). Out of all the NMD factors, the levels of SMG1 expression was among the strongest predictors of T-cell immune infiltration in the three cohorts. Thus, we chose SMG1 expression as the basis of stratifying patients into two groups consisting of low SMG1 and high SMG1 tumors (16 low SMG1 and 15 high SMG1 for BRCA, 12 low SMG1 and 12 high SMG1 for PDAC, 6 low SMG1 and 5 high SMG1 for LUAD). Subsequently, dimensionality reduction with uniform manifold approximation and projection (UMAP) was performed, followed by the separation of tumor samples based on high and low tumor-SMG1 expression in the tumor cells. The group of patients with lower SMG1 levels were characterized by higher T-cell immune infiltration in BRCA (Fig. 1D, E), PDAC (Fig. S1B, C) and LUAD (Fig. S1E, F). In LUAD and PDAC there was also a significant increase in B lymphocytes in the patients with low SMG1 expression.

As the BRCA study [21] included the detailed scRNAseq immune dataset, we performed a precise analysis of the different T-cell subpopulations after dimensional reduction (UMAP) and separation in the group of patients based on low SMG1 and high SMG1 expression in the tumor cells. This study reflected that low SMG1 tumor expression shows higher signs of T-cell activation with more CD8 effector memory function. Tumor samples with higher SMG1 expression levels displayed more abundance of CD8 naïve phenotype while all the other T-cell subtypes were similar or much more abundant in the SMG1-low tumors (Fig. 1F, G). In association with higher T-cell activation in SMG1-low tumors, we also observed a deeper T-cell exhaustion phenotype (Fig. 1F, G). Even when patients were stratified in quartiles depending on SMG1 expression in tumor samples, those with lower tumor levels of SMG1 displayed a CD8 phenotype with more abundance of cells expressing exhaustion markers (LAG3, PD-1, CTLA-4, TIM3, TOX) (Fig. 1H).

### NMD activity affects tumor growth in an immune-dependent manner

**Fig. 2:**
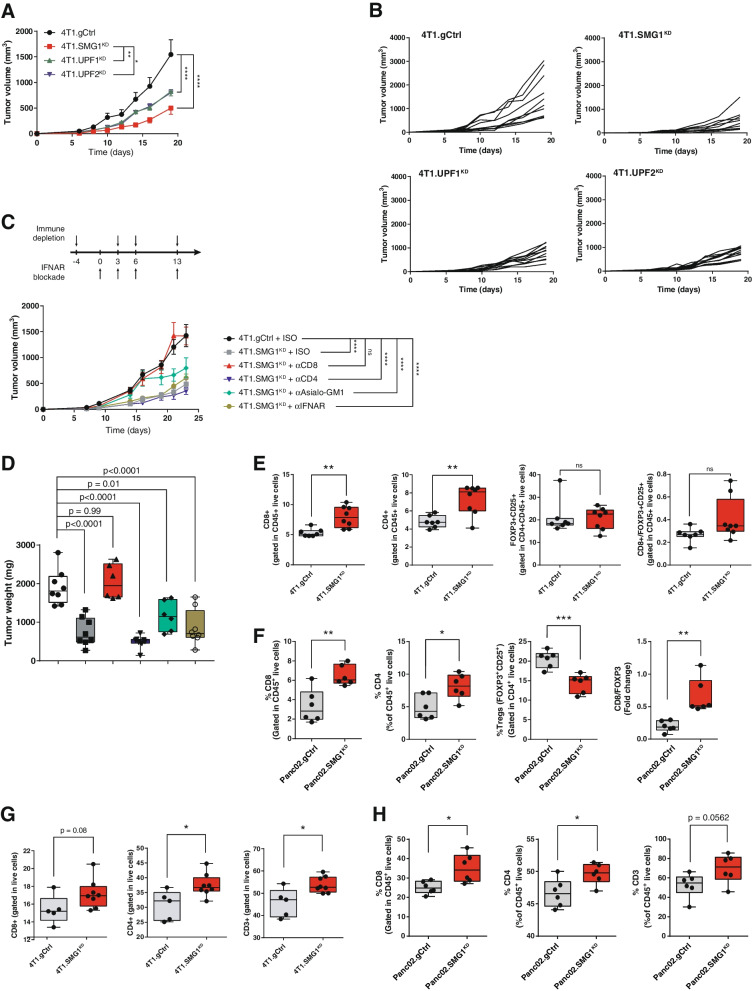
SMG1 reduction in mouse breast cancer tumor cells slow tumor growth in an immune-dependent manner. (**A**) Several NMD factors (SMG1, UPF1 and UPF2) were knocked-down using CRISPR/Cas9 in 4T1 breast cancer mouse model implanted subcutaneously in Balb/c mice and tumor growth was measured over time. *n* = 10-12/group. (**B**) Tumor volumes from (A). Each line depicts the growth over time of an individual tumor. *n* = 10-12/group. (**C**) Top: Experiment schedule. Bottom: Tumor volume over time of 4T1.gCtrl tumors implanted subcutaneously in Balb/c mice treated with isotype, anti-CD4 (clone GK1.5), anti-CD8a (clone 53-6.7), anti-Asialo GM1 (Poly21460) (NK) depleting or anti-IFNAR blocking antibodies (clone MAR1-5A3). *n* = 6-8/group. (**D**) Tumor weights of (C) at end point (day 24). *n* = 6-8/group. (**E**) Knockdown of SMG1 in 4T1 cells enhances immune infiltrate. Balb/c mice were subcutaneously implanted with 4T1.gCtrl or SMG1^KD^ cells and on day 14 tumors were resected and analyzed by flow cytometry. *n* = 7-8/group. (**F**) Knockdown of SMG1 in Panc02 cells enhances immune infiltrate. C57/BL6 mice were subcutaneously implanted with Panc02.gCtrl or SMG1^KD^ cells and on day 14 tumors were resected and analyzed by flow cytometry. *n* = 6/group. Left to right: CD8 T cell infiltration, CD4+ T cell infiltration, Treg (FOXP3+CD25+) T-cell infiltration, CD8+/Treg ratio. *n* = 6/group. (**G**) Draining lymph node flow immune analysis by cytometry study in 4T1.SMG1^KD^ and 4T1.gCtrl tumor bearing mice at day 14. *n* = 5 (4T1.gCtrl group); 8 (4T1.SMG1^KD^ group). (**H**) Draining lymph node flow immune analysis by cytometry study in Panc02.SMG1^KD^ and Panc02.gCtrl tumor bearing mice at day 14. *n* = 6/group. p-values are shown for 2-way ANOVA with Bonferroni’s post-hoc test for tumor growth experiments. 1-way ANOVA was performed in (D); and 2-tailed t-test was performed in the infiltrate study. ∗*p* < 0.05, ∗∗*p* < 0.01, ∗∗∗*p* < 0.001

To recapitulate the effect of NMD activity in the outcome of tumor progression, we generated different tumor cell lines with their NMD machinery compromised by blocking key NMD factors -SMG1, UPF1, UPF2- via CRISPR. The mutation in the target gene was sequenced by Sanger sequencing and confirmed via Tracking of Indels by DEcomposition (TIDE) (Fig. S2A-C) and western blot when antibody was available (Fig. S2D). In addition, we used an NMD reporter plasmid to check that NMD activity was successfully downregulated in the generated cell lines (Fig. S2E). We checked that genomic edition of SMG1 persisted a minimum of 3 months in culture (Fig. S2F). To reduce the effect of genetic drift that can lead to artificial differences in tumor growth *in vivo*, we chose to work with a pool of CRIPSRed tumor cells with a heterogeneous range of indel mutations in the target gene (Fig. S2A-C, right panels) where some might not lead to a complete gene disruption. However, we ensured a substantial reduction of the target protein (Fig. S2D). As a control, we also included a tumor clone derived from a single tumor cell by dilution after sorting, containing a unique type of CRISPR-mutation in SMG1 gene as well as complete SMG1 blockade in the B16/F10 cell line. Possible CRISPR off-target artifacts in other parts of the genome were ruled out by sequencing the top 2 to 3 predicted sequences with some homology to the sgRNA. As SMG1 is the first factor to initiate NMD activation and showed a high-predicted value of tumor survival and inflammation (Fig. 1) we generated SMG1^KD^-derived cells from 4T1 breast cancer, Panc02 for Pancreatic Adenocarcinoma and B16/F10 as Melanoma murine models. UPF1 and UPF2 were also gene edited in 4T1 and B16/F10 tumor. Even though melanoma was not one of the top tumor types that crosslink survival and SMG1 expression in TCGA, we chose to include this model as well to evaluate whether future pharmacological NMD inhibition could be translated onto a broader range of tumors. We monitored the growth of SMG1 genetically inhibited tumors (SMG1^KD^) implanted in syngeneic mice. In all cases, we observed a significant reduction of tumor progression of SMG1^KD^ tumors compared to the controls. The inhibition was also observed when other NMD factors were genetically inhibited via CRISPR (UPF2 or UPF1), but to a lower extent than observed with SMG1 blockade (Fig. 2A, B; Fig. S3A-D). Interestingly, the tumors derived from B16/F10 clone with complete SMG1 disruption displayed higher antitumor response (Fig. S3B, D). Importantly, inhibition of SMG1 factor did not show any deleterious effect in vitro with similar cell growth kinetics observed in all the cell lines in culture (Fig. S2G) after 3 weeks. The antitumor response of NMD inhibition was dependent on the immune system, as tumor kinetics of 4T1 SMG1^KD^ was similar to the control when implanted in immunodeficient mice (Rag2/IL2rg^-/-^) (Fig. S3E-F). For further validation of the importance of the different immune cells in the antitumor response in the SMG1^KD^ setting, we selectively depleted the different immune cell compartment *in vivo* using immuno-ablative antibodies (anti-CD8a, anti-CD4 and anti-Asialo-GM1). We also blocked the IFN-α axis by using an IFNAR blocking antibody to rule out innate immune system dependent responses mediated by IFN-α. The abrogation of the antitumor response was clearly dependent on CD8 lymphocytes, and a minimal effect at early stages was observed with NK depletion with anti-Asialo-GM1 as well (Fig. 2C, D). Depletion of the immune populations of interest was checked *in vivo* by flow cytometry (Fig. S3G-H).

We also performed an analysis of the immune infiltrate in SMG1^KD^ tumors and in the draining lymph nodes of 4T1 and Panc02 models. We detect an increased frequency of T lymphocytes in SMG1^KD^ tumors, especially CD8 cells in the tumor milieu (Fig. 2E, F) as well as in the tumor draining lymph nodes (Fig. 2G-H). Data was analyzed using the gating strategy shown in Fig. S4.

As we observed that tumor samples with low SMG1 expression were more abundant in exhausted CD8 lymphocytes (Fig. 1F-H) we decided to evaluate whether the inhibition of NMD could improve the outcome of anti-CTLA-4 and anti-PD-1 (ICB) therapy in 4T1 tumors. The inhibition of SMG1 displayed an antitumor effect similar to that of blockade with anti-CTLA-4 and anti-PD-1 antibodies, and when ICB was combined with SMG1 inhibition the antitumor effect improved (Fig. S3I).

### Tumors with reduce SMG1 expression display alternative TCR repertoires with higher rate of immune responses to neoantigens containing frameshift mutations with PTCs

**Fig. 3:**
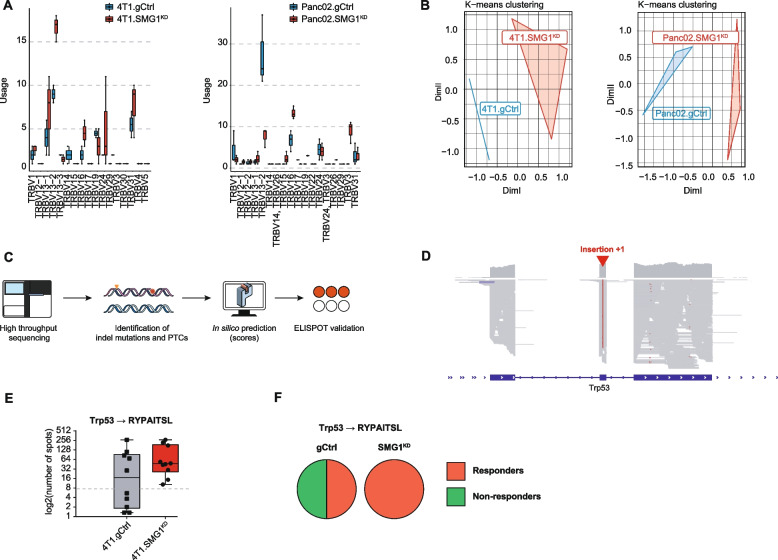
SMG1 expression affects the TCR repertoire and conditions PTC-neoantigen immune responses. (**A**) Panc02 or 4T1 (gCtrl or SMG1^KD^) tumor-bearing mice are sacrificed on day 14 post-inoculation and tumor draining lymph node mRNA is isolated for TCRseq. Abundance of the TRBV segment present in the most frequent clones (>50 copies) was analyzed. *n* = 3/group (Panc02.SMG1^KD^); n = 3/group (Panc02.gCtrl); *n* = 3/group (4T1.SMG1^KD^); *n* = 2/group (4T1.gCtrl). (**B**) K-means t-sne clustering of TRBV segment from (A). *n* = 3/group (Panc02.SMG1KD); *n* = 3/group (Panc02.gCtrl); *n* = 3/group (4T1.SMG1^KD^); *n* = 2/group (4T1.gCtrl). (**C**) Pipeline followed for neoantigen discovery in 4T1 mouse breast model. (**D**) Integrated Genome Browser (IGV) view of the +1 insertion in Trp53 present in 4T1 cells. (**E**) ELISPOT determining NMD-controlled immune response induction of p53 neoantigen in 4T1 tumor-bearing mice. Balb/c mice were sacrificed on day 14 post-inoculation of gCtrl or SMG1^KD^ 4T1 tumors and draining lymph nodes were isolated. Obtained cells were co-cultured with the candidate peptides to test antigen recognition. *n* = 10/group. (**F**) Proportion of responding and non-responding mice to Trp53 neoantigen in (E). All SMG1^KD^ tumor-bearing mice show response to Trp53 peptide compared to the 50% of gCtrl group

Given the associations of NMD and tumor immune infiltration, we next explored changes in the TCR repertoire of the T cells in the draining lymph nodes of SMG1^KD^ tumor-bearing mice. Changes in the TCR clone repertoire induced by NMD inhibition could be a consequence of reshaping of the tumor antigen landscape. To that end, we performed bulk CDR3 TCRBV sequencing. Considering the top 50 clones enriched in each sample, we determined the frequency of TCRBV segments present in the bulk T cells. We observed changes in the frequencies of TCRBV rearrangements in the lymphocytes derived from SMG1^KD^ tumor-bearing mice compared to the controls, indicating changes in antigen immunodominance upon NMD inhibition (Fig. 3A). Importantly, the TCRBV segments from SMG1^KD^ tumor-bearing mice clustered together and not with control tumors in the 4T1 Balb/c and Panc02 C57/BL6 murine models (Fig. 3B).

Next, we wanted to identify potential frameshifts in the transcriptome that could lead to neoantigens under NMD control. Thus, we performed full length RNAseq of 4T1 tumors and identified different transcripts with potential changes in the frameshifts (Fig. 3C) including a mutation in Trp53 that leads to a nucleotide insertion in the exon 2 (Fig. 3D and S5A-B) that causes a frameshift with the appearance of PTC (Fig. S5C). The list of potential neoantigen peptides derived from frameshifts in the transcriptome were run through netMHCpan pipeline to predict high score peptides loaded in MHC-I (H2Kd, H2Dd, H2Ld). The ones with higher score were synthesized to assert the specific immune response in mice implanted with 4T1 SMG1^KD^ tumors. Out of all the peptides tested, the one derived from Trp53 (RYPAITSL) was the most efficiently recognized by the immune cells on SMG1^KD^ 4T1 tumor-bearing mice. Interestingly, when we tested the immune response against RYPAITSL in mice implanted with 4T1.gCtrl tumors, we observed that few of them also mounted a strong response against this neoantigen (Fig. 3E, F and S5D). All mice implanted with SMG1^KD^ 4T1 tumors responded against RYPAITSL while in 4T1.gCtrl tumor-bearing mice the rate of response was 50%. The partial response to RYPAITSL in 4T1.gCtrl tumor bearing mice could be explain if NMD activity is not completely efficient at eliminated PTC-mRNA, considering this possibility we performed a prophylactic vaccine experiment using whole tumor vaccines with SMG1^KD^ cells or the cognate control cell lines. We injected both in a suboptimal dose of tumor cells that cannot engraft and progress into a tumor or irradiated whole tumor cells as a platform of immunization (Fig. S5E-F). gCtrl tumors (Panc02 in C57/BL6 or 4T1 for Balb/c) were implanted after the immunization protocol and tumor kinesis was monitored. The only vaccine that elicited an antitumor response delaying tumor growth was that from SMG1^KD^ cells (Fig. S5E-F). The immune response elicited against SMG1^KD^ tumors still recognize the 4T1.gCtrl tumors reassessing the possibility that the neoantigen under NMD control can be leaky [10, 24, 25] and thus potentially regulated.

### SMG1 expression fluctuates over the course of the ICB treatment in association with T-cell expansion in breast cancer patients

**Fig. 4:**
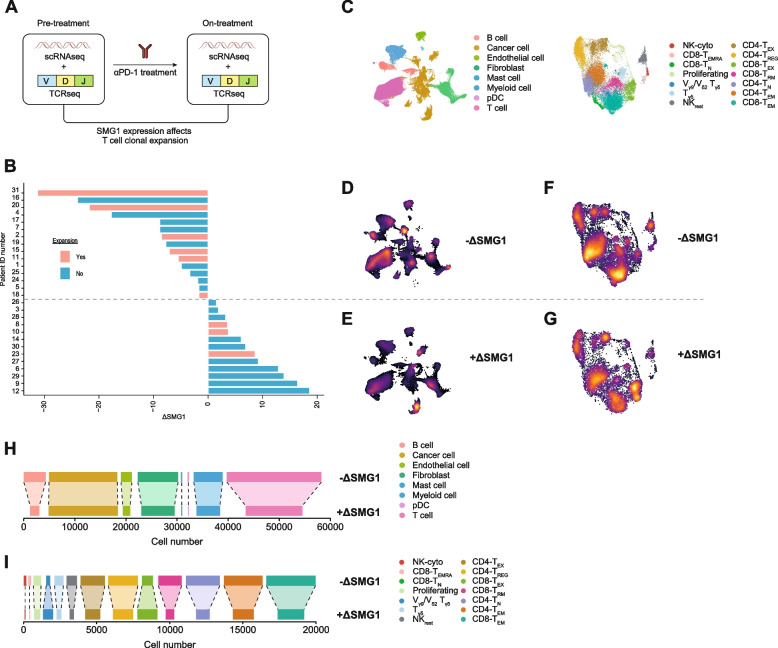
SMG1 upregulation after anti-PD-1 therapy associates with reduced immune infiltration and compromised T-cell expansion in breast cancer patients. (**A**) Study design from [21] used to interrogate whether SMG1 expression fluctuation during anti-PD-1 treatment affect at TCR clonal expansion in breast cancer patients (*n* = 28) (**B**) Fluctuation of SMG1 expression (ΔSMG1) in breast cancer patient during the course of anti-PD1 treatment. Patients determined with positive TCR clonal expansion after anti-PD1 treatment are depicted in red. (**C**) Legend for the different cell types assigned in the UMAP clusters. (**D**) Represent the UMAP intensity of the group of patients with reduction on SMG1 expression after anti-PD-1 treatment (-ΔSMG1) (**E**) Represent the UMAP intensity of the group of patients with enhance SMG1 expression after treatment (+ΔSMG1). (**F**) Represent the UMAP intensity from the tumor immune infiltrate of the group of patients with reduction on SMG1 expression after anti-PD-1 treatment (-ΔSMG1). (**G**) Represent the UMAP intensity from the tumor immune infiltrate of the group of patients with enhance SMG1 expression after treatment (+ΔSMG1). (**H**) Bar plot showing absolute cell numbers of each population shown in (D-E). (I) Bar plot showing absolute cell numbers of each population shown in (F-G)

In line with the hypothesis that NMD might function as checkpoint of tumor antigenicity, we interrogated the scRNAseq dataset from the study [21] to see if higher expansion of T cells could be associated with SMG1 changes upon anti-PD-1 antibody treatment (Pembrolizumab). This study contains scRNAseq data from pre-treatment tumor samples (pre) and on-treatment (on) tumor samples from breast cancer patients being treated with Pembrolizumab (Fig. 4A). From the same patients, we gathered information on the frequency of TCR rearrangements, which allowed us to determine the clonal expansion induced by anti-PD-1 blocking antibodies. We considered BRCA patients with positive expansion upon anti-PD-1 treatment with p-values lower than 0.002 (Fig. S6A). Given the potential role of SMG1 in tumor immunity and the fact that SMG1 expression varies in tumor samples, we stratified the patients based on changes in SMG1 expression (ΔSMG1) after anti-PD-1 antibody treatment (Pembrolizumab). Bearing in mind this criterion, the tumor samples from patients that upregulated SMG1 expression (+ΔSMG1) after treatment with Pembrolizumab were characterized by a lower clonal expansion. Only three patients with positive clonal expansion had increased levels of SMG1 upon treatment (Fig. 4B); interestingly, two of those patients (8 and 10) are precisely the ones with lower levels of SMG1 in pre-treatment tumor samples (Fig. S6B). To corroborate the TCR clonal expansion we also analyzed by dimensionality reduction (UMAP) each tumor sample separately in pre and on treatment from the scRNAseq BRCA dataset (Fig. S6C and S6D). All patients (6/6) with positive clonal TCR expansion after anti-PD-1 treatment with reduce level of SMG1 expression (-ΔSMG1) showed also higher T cell infiltration as compare to the pretreatment stage (Fig. S6C, left panel). In case of the group of patients with upregulation of SMG1 (+ΔSMG1) after ICB treatment, patient 8 in spite of considered as positive for TCR clonal expansion showed a reduction on T cell infiltrate after the ICB treatment (Fig. S6D, left panel) indicating that the TCR clonal expansion of patient 8 might be overestimated. In sum, this result suggest that high basal level of SMG1 expression in the tumor in pre-treatment as well as upregulation of SMG1 (+ΔSMG1) after ICB might restricts the expansion of T cells infiltration in the tumor.

Apart from T-cell expansion we evaluated the nature of immune infiltrates in tumor samples after treatment (Fig. 4C). In line with T-cell expansion, we observed that tumors that upregulate SMG1 (+ΔSMG1) expression were characterized by a lower amount of T and B lymphocytes in the tumor milieu (Fig. 4D, E and H). After dimensional reduction on scRNAseq immune T-cell sub-clustering, we were able to identify the different subpopulations of lymphocytes that infiltrate the tumor (Fig. 4F, G). Remarkably, we observed that the group of tumors with SMG1 upregulation (+ΔSMG1) upon treatment with anti-PD-1 displayed a lower amount of CD8 effector memory cells and a considerable increase in exhausted CD8 and CD4 lymphocytes (Fig. 4F, G and I).

### Tumors escape the immune response against frameshift-derived neoantigens in an SMG1-dependent manner

**Fig. 5:**
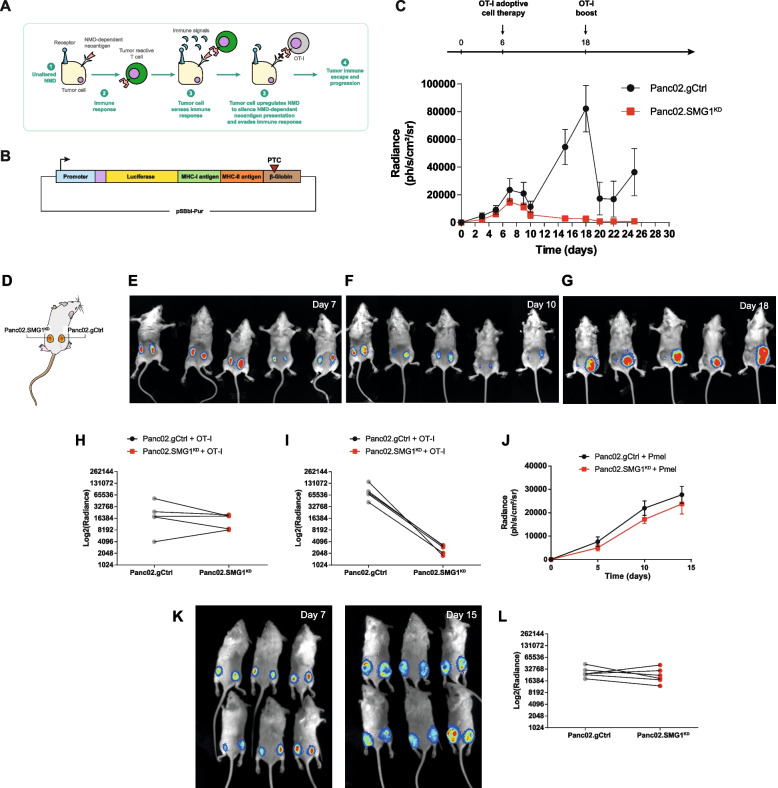
Tumors upregulate NMD as an immune-escape mechanism to suppress PTC-containing antigen presentation. (**A**) Scheme depicting the tumor mechanism of immune evasion by NMD upregulation to silence NMD-controlled neoantigens. (**B**) (A) Scheme of our NMD luciferase-SIINFEKL plasmid construct. Elements cloned in the plasmid (from left to right): EF-1α promoter (blue); PEST motive (violet); luciferase (yellow); SIINFEKL cDNA (green); DDCWFYFTYSVNGYNNEAIVHVVETPDCP MHC-II peptide [26], flanked by 2 cathepsin sites; β-Globin PTC-39 cassette (brown). (**C**) Top: Experiment schedule. Bottom: Rag2/IL2rg^-/-^ mice were injected with Panc02.gCtrl (right flank) or SMG1^KD^ (left flank) cells expressing luciferase-SIINFEKL reporter plasmid and radiance was measured over time. On day 6, 8 x 10^6^ activated OT-I splenocytes were transferred intravenously. On day 18, 50 μg of SIINFEKL peptide was administered intraperitoneally to induce OT-I reactivation. *n* = 5/group. (**D**) Scheme depicting Panc02.gCtrl injected in the right flank and SMG1^KD^ in the left one. (**E**) Images of Rag2/IL2rg^-/-^ mice from (B) on days 7, (**F**) Day 10 and (**G**) Day 18. (D) Radiance comparison between gCtrl and SMG1^KD^ on day 7 from (B) post-tumor inoculation. *n* = 5/group. (**H**) Radiance comparison between gCtrl and SMG1^KD^ on day 18 from (B) post-tumor inoculation. n = 5/group. (G) Radiance of Panc02 tumors from (B) on day 7 comparing gCtrl vs. SMG1^KD^. (**I**) Radiance of Panc02 tumors from (B) on day 18 comparing gCtrl vs. SMG1^KD^. (**J**) Rag2/IL2rg^-/-^ mice were injected with Panc02.gCtrl (right flank) or SMG1^KD^ (left flank) cells expressing luciferase-SIINFEKL reporter plasmid, radiance was measured over time. On day 6, 8 x 10^6^ activated Pmel splenocytes were transferred intravenously. Since SIINFEKL recognition cannot occur, no luciferase changes were observed. *n* = 6/group. (**K**) Caption of mice on day 7 (left) and 15 (right) from (J). (**L**) Radiance comparison between gCtrl and SMG1^KD^ on day 15 from (J) post-tumor inoculation. *n* = 6/group

We hypothesize that NMD is upregulated by intrinsic inflammatory signal that are indirectly dependent on the immune infiltrate (Fig. 5A). To gain further insights into the process of NMD regulation, we generated a reporter platform based on the expression of a firefly luciferase reported construct cloned upstream of a PTC susceptible to NMD degradation; together with luciferase we included a potent antigen determinant presented by MHC class I (SIINFEKL) and class II (DDCWFYFTYSVNGYNNEAIVHVVETPDCP) [26] (Fig. 5B). It can be argued that SIINFEKL is an artificial non-encoded protein in the mouse genome, but precisely this is the type of strong “immune-foreign” neoantigen induced by frameshift mutations in which the sequence of the protein changes completely from the mutation site. This NMD reporter plasmid was used to transfect tumor cells. From our observations (Fig. 3E, F), NMD is a not absolute efficient process, thus opening the possibility of being regulated. In this scenario, the expression of NMD-targeted protein (potential neoantigen) will depend on the balance of transcription and NMD activity (Fig. S7A). Using this luciferase-PTC reporter plasmid, we can visualize this balance through luminescence-based assays. To assess this possibility, we transfected tumor cells Panc02.SMG1^KD^ and control cells with different ratios of the NMD reporter plasmid (Fig. S7B). As expression of the reported plasmid increased, NMD was not able to cope with the number of newly PTC-transcripts, and thus, there was an overflow of antigen leading to a higher leakage from NMD and triggering stronger OT-I activation (Fig. S7B). Panc02.SMG1^KD^ tumors, as expected, were not able to degrade any of the PTC-containing antigen, and thus triggered a stronger OT-I activation response. Based on the transfection conditions we can generate tumor stable cell lines that induce similar levels of luciferase (Fig. 5C, E) and similar OT-I lymphocytes responses in Panc02.gCtrl and Panc02.SMG1^KD^ tumor cells despite NMD disruption (Fig. S7C).

To assess whether SMG1 expression can condition the outcome of immune responses, we implanted tumor cells with similar OT-I activation capability (as measured *in vitro*) (Fig. S7C) in the same mice but on their opposite flanks. To isolate the effect of the immune response against a specific antigen and interrogate the mechanisms of immune escape mediated by NMD, we performed the experiment in immunodeficient mice (Rag2/IL2rg^-/-^) that were adoptively transferred with activated OT-I lymphocytes post tumor implantation and quantified for their luciferase signal along the course of the experiment (Fig. 5C-G). As expected, the level of luciferase that correlates with the amount of SIINFEKL was similar in both tumor flanks of the implanted mice at early time points (Fig. 5C, E and H). As soon as the mice were adoptively transferred with activated OT-I lymphocytes, the luciferase signal decayed drastically in both tumors (Fig. 5C and F). In the case of SMG1^KD^ tumors, the luciferase signal was completely abrogated, while in the case of control tumors, the luciferase inhibition was transient, bouncing back a few days after the adoptive transfer of OT-I lymphocytes (Fig. 5C, G and I). The re-activation of OT-I lymphocytes by SIINFEKL boost reduced the luciferase signal, again transiently, but the tumor continued to escape the antigen-specific immune pressure (Fig. 5C). Importantly, when mice were co-implanted with both SMG1^KD^ and control tumors and transferred with non-tumor-reactive lymphocytes (Pmel), the luciferase signal was not affected in any of the tumors (Fig. 5J-L).

To ensure that the SMG1 inhibition was not affecting the immunogenicity of the tumor by other mechanisms not directly related to the antigen expression under NMD control, we first confirmed that MHC-I expression was not altered in SMG1^KD^ tumors (Fig. S8A). Second, we performed a functional assay in which Panc02.SMG1^KD^ or Panc02.gCtrl cells were exogenously loaded with increasing doses of SIINFEKL peptide, and after washed, they were used to prime OT-I lymphocytes in vitro (Fig. S8B). The immune response generated by OT-I lymphocytes under these conditions was similar to that generated using SMG1^KD^ and control tumors, confirming that the higher immunogenicity of the SMG1^KD^ tumors mainly depends on intrinsic stabilization of the antigen that is under NMD control (Fig. S8C). The final validation was done *in vivo*, in experiments where we eliminated the PTC from the antigen-luciferase reporter plasmid, and we conducted a similar experiment involving adoptive transfer of OT-I lymphocytes in contralaterally implanted mice bearing SMG1^KD^ and Panc02.gCtrl tumors. The absence of a PTC eliminated the checkpoint function of NMD and both tumors were similarly controlled by the transfer of OT-I lymphocytes *in vivo* (Fig. S8D-F).

**Fig. 6:**
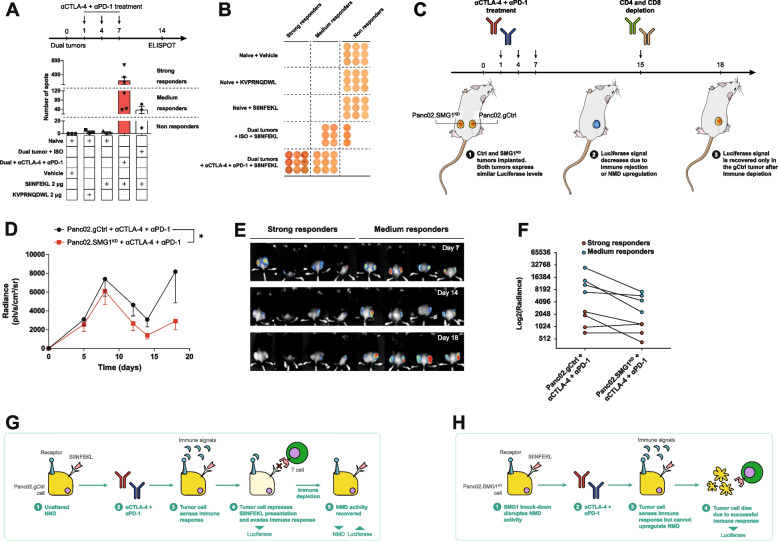
Tumors upregulate NMD to evade ICB therapy induced immune response. (**A**) Top: treatment schedule. C57/BL6 mice were implanted with Panc02.gCtrl on the right flank and SMG1^KD^ on the left flank. Both cell lines expressed the NMD reporter plasmid that contains SIINFEKL under the control of a PTC, mimicking a tumor antigen under NMD pressure. Anti CTLA-4 + anti PD-1 combination treatment was injected intraperitoneally as indicated. Bottom: On day 14 mice were sacrificed and cells from tumor-draining lymph nodes were isolated and IFN-γ ELISPOT assay against SIINFEKL peptide was carried out. Human gp100-derived peptide KVPRNQDWL was used as negative control. n = 3-6/group. (**B**) Individual ELISPOT wells from (A) classified in strong, medium or non-responders. (**C**) Treatment schedule of (**D**). (D) C57/BL6 mice were implanted with Panc02.gCtrl (right flank) or SMG1^KD^ (left flank) cells expressing luciferase-SIINFEKL reporter plasmid and radiance was measured over time. Mice were treated with 100 μg of each: anti CTLA-4 and anti PD-1 antibodies (A). On day 15 we depleted CD8 and CD4 T cells by injecting CD4 and CD8 antibodies (200 μg of each, clones GK1.5 and 53-6.7 respectively). *n* = 7. (**E**) Images showing luciferase intensity on days 7, 15 and 18. (F) luciferase levels on day 18 comparison between control tumor (left) and SMG1^KD^ (right) from (**F)**. p-values are shown for 2-way ANOVA with Bonferroni’s post-hoc test. ∗*p* < 0.05, ∗∗*p* < 0.01, ∗∗∗*p* < 0.001. (**G**) Mechanism proposed for the luciferase changes observed in anti-CTLA-4 + anti-PD-1-treated Panc02.gCtrl tumors. ICB therapy elicits a strong immune response and is sensed by the tumor cell. Cell with unaltered NMD trigger upregulation of NMD (reflected in a decrease of luciferase signal) in tumors to repress the presentation of PTC-controlled antigens (SIINFEKL in our scenario) by degrading their mRNAs via this surveillance mechanism. When the immune response is evaded by tumor cells, they recover normal NMD activity which we detected as a luciferase signal increase. (**H**) Mechanism proposed for anti-CTLA-4 + anti-PD-1-treated mice bearing NMD-reporter-expressing Panc02.SMG1^KD^ tumor cell line. In contrast with control cells (C), SMG1^KD^ cells are unable to modulate NMD activity. In this case, the immune response is capable of eliminating SIINFEKL^+^ tumor cells

Next, we aimed to evaluate whether this process could also take place under other immune pressures such as those appearing under ICB therapy (anti-PD-1 and anti-CTLA-4). For this purpose, we used the NMD reporter cell lines previously described (Fig. 5 and S7). As the immune response in wild type mice may be more heterogeneous due to polyclonality, we wanted to confirm that under our experimental conditions, the antigen (SIINFEKL) expressed in the reporter plasmid was eliciting an immunodominant response that could be detected. To this end, mice contralaterally implanted with both Panc02.SMG1^KD^ and Panc02.gCtrl tumors were monitored for the induction of an immune response against SIINFKEL in the absence or presence of ICB treatment (Fig. 6A). The grade of the immune response against SIINFEKL, as expected, was heterogeneous, but those mice treated with anti-PD-1 and anti-CTLA-4 mounted strong to medium immune responses, as measured by IFN-γ ELISPOT (Fig. 6A). On the other hand, tumor-bearing mice treated with the isotype control antibody mounted a considerably weaker immune response against SIINFEKL (Fig. 6A, B). In light of this, we decided to evaluate luciferase signal as a measure of the efficacy of the antitumor immune response triggered by anti-PD-1 and anti-CTLA-4 treatment. Mice were implanted with both tumors (Panco02.SMG1^KD^ and Panc02.gCtrl) expressing similar levels of luciferase and SIINFEKL (Fig. 6D, S7C) and treated with anti-PD-1 and anti-CTLA-4 at days 1, 4 and 7 post tumor inoculation (Fig. 6C). To track any possible dependency of the immune response on the regulation of NMD and counteract an excessive immune response, we depleted T lymphocytes (CD8 and CD4) on day 15. Luciferase signal was monitored at different time points. Initially, until the adaptive immune response was mounted (circa day 8), both tumors showed increased luciferase signal in a similar manner (Fig. 6D and E). From day 8 until day 15, both tumors were targeted by the antitumor immune response with a decay in their observed luciferase signal (Fig. 6D and E). However, as soon as the immune pressure was eliminated by T-cell depletion, many control tumors rapidly recovered their luciferase signal while SMG1^KD^ tumors did not. This phenomenon was probably more relevant in mice that triggered a moderate antitumor immune response than on those with stronger antitumor immune responses. This can be explained because NMD (Fig. 3E, F, S5E-F and S7C) is not complete and, even if it were upregulated by some type of signal related to tumor inflammation, there might still be some antigen leakage that leads to tumor recognition when there is a very strong effector immune response.

### SMG1 is upregulated in an inflammatory context by the IL-6/STAT3 axis, silencing frameshift-derived neoantigens

**Fig. 7:**
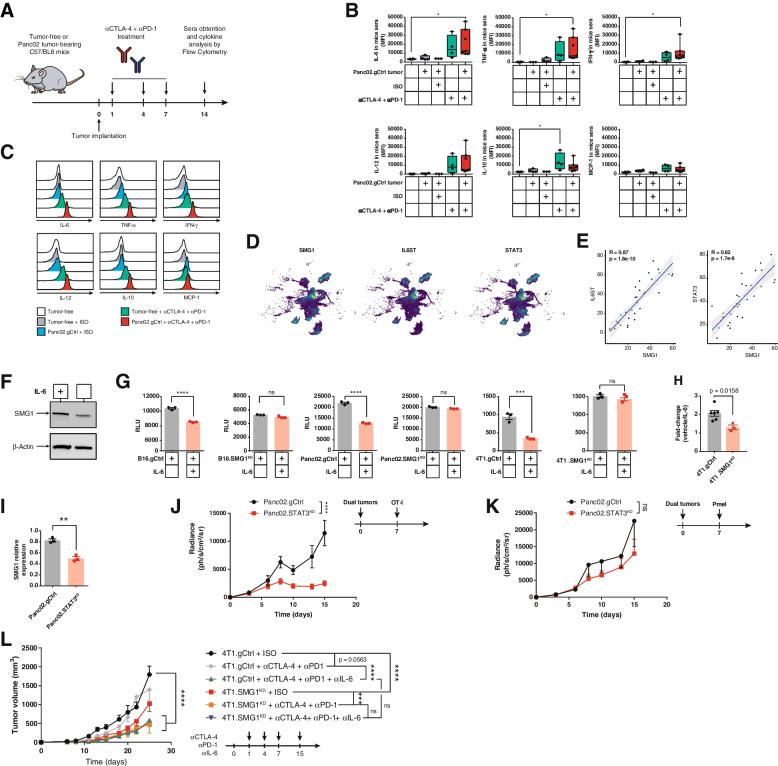
IL-6 pathway is activated in tumors treated with ICB therapy upregulating NMD activity. (**A**) Treatment schedule for (A) and (B). (**B**)Tumor-free and Panc02.gCtrl tumor-bearing C57/BL6 mice were treated with isotype control (ISO) SO or anti-CTLA-4 + anti-PD-1 combination or untreated on day 1, 4 and 7 and n day 14 post tumor inoculation, mice were bled, to analyze TNF-α, MCP-1, IL-12, IL-10, IL-6 and IFN-γ by Cytometric Bead Array (CBA). (B) Cytokine levels measured by CBA in mouse sera from (A). n = 3-7/group. (**C**) Representative individual sample from (B) for each cytokine. (**D**) UMAP depicting expression of SMG1, IL6ST and STAT3 in tumor cells. IL-6 signaling factors perfectly co-localize with SMG1 [21]. Only tumor cells are shown in this figure. (**E**) IL6ST (gp130) and STAT3 expression at scRNAseq in malignant cells from (D) show a highly significant linear correlation with SMG1. (**F**) IL-6 signaling upregulates NMD expression in B16.gCtrl cell line. B16.gCtrl were incubated in the presence of hyper-IL-6 for 96 h. Protein levels were analyzed by western blot. (**G**) IL-6 signaling upregulates NMD. B16, Panc02 and 4T1 mouse tumor cells stably transduced with our luciferase-SIINFEKL NMD reporter plasmid were plated and murine hyper-IL-6 or vehicle was added to the media. 96 h later, luciferase signal was measured. *n* = 3. (**H**) 4T1.gCtrl or SMG1^KD^ were treated as in (F). Trp53 mRNA levels were measured by qRT-PCR. (**I**) 4T1.gCtrl and STAT3KD were treated with hyper-IL-6 as in (F). SMG1 mRNA levels were measured by qRT-PCR. (**J**) STAT3^KD^ or gCtrl Panc02 were injected in the left and right flanks, respectively, of Rag2/IL2rg^-/-^ mice. Activated OT-I splenocytes were administered intravenously as shown in the schedule (right). *n* = 9. (**K**) STAT3^KD^ or gCtrl Panc02 were injected in the left and right flanks, respectively of Rag2/IL2rg^-/-^ mice. Activated Pmel splenocytes were administered intravenously as shown in the schedule (right). *n* = 7. (**L**) Antitumor effect of IL-6 blockade and NMD knockdown. Balb/c mice were injected with 4T1.gCtrl or SMG1^KD^ cells. Treatment was carried out as shown in the tumor schedule. *n* = 6-8/group. 2-way ANOVA corrected with Bonferroni’s test was performed for tumor growth and luciferase evolution over time; 1-way ANOVA corrected by Bonferroni’s test was used in (B); 2-tail t-test employed in (F). ∗*p* < 0.05, ∗∗*p* < 0.01, ∗∗∗*p* < 0.001. ns = non-significant (*p* > 0.05)

Bearing in mind all the data indicating that NMD plays an important role in antigen immune escape, with the SMG1 factor as one of initial inducers of the pathway, we considered that SMG1 might be a checkpoint regulated by some inflammatory signals released upon the initiation of the antitumor immune response (Fig. 6G and H; Fig. S9A-B). To address this possibility, we measured a panel of relevant inflammatory cytokines that can be produced by anti-CTLA-4 and anti-PD-1 treatment and by adoptive transfer therapy of tumor-reactive lymphocytes. We observed that the simple implantation of control tumors in immunocompetent mice triggered several inflammatory cytokines in sera, but when the mice were also treated with anti-CTLA-4 and anti-PD-1 antibodies the levels of IL-6 and TNF-α were further induced (Fig. 7A-C). Interestingly, during the adoptive transfer of activated OT-I lymphocytes into tumor-bearing mice, the cytokine that was more highly induced was IL-6 (Fig. S10A-C).

Given the fact that IL-6 was one of the most induced cytokines in the context of cancer immunotherapy (ICB and adoptive therapy), we wanted to evaluate whether the IL-6/STAT3 axis could in fact be upregulating SMG1. As an initial approach to assess whether both pathways were intertwined, we analyzed scRNAseq datasets form breast cancer [21], Pancreatic cancer [23] and Lung Adenocarcinoma [22]. Looking into the expression of SMG1, IL6ST (IL-6 signal transducer) and STAT3 in single tumor cells from those datasets, we observed that higher expression of SMG1 co-localized in the same cells that had higher levels of IL6ST, being such association very strong with the STAT3 factor as well (Fig. 7D and S10D-E). Next, we looked into the average expression of SMG1, STAT3 and IL6ST only in tumor cells from each patient and we performed a correlation analysis for the expression or each factor with SMG1. BRCA patients showed a striking correlation (Fig. 7E). In the case of PDAC patients, the positive correlation was significantly high with SMG1/STAT3 (Fig. S10F). LUAD dataset includes only a few patients, which might explain the weaker correlations (Fig. S10F). To validate this hypothesis, we analyzed the expression of SMG1 and STAT3 factors by qRT-PCR in a repertoire of human tumor cell lines (29 in total, of different origins, including many lung cancer cell lines), that were selected for their positiveness for IL-6 production. The correlation of SMG1 and STAT3 in the cell lines was extremely high, indicating that STAT3 transcription factor might be involve in SMG1 expression (Fig. S10G, Table S1). Using a STAT3 ChIP-seq dataset from [27] we confirmed that STAT3, can in fact, bind to SMG1 promoter, indicated by the presence of a MACS peak (Fig. S10H). This was corroborated in the UCSC Genome Browser for STAT3 reported binding sites (Fig. S10I). Given the potential role of IL6ST/STAT3 axis in SMG1 regulation and the important role it might play in tumor antigenicity we interrogated the RNA expression data from TCGA repository so as to identify immune pathways that could be associated with the expression of SMG1. For each tumor type in the TCGA, we established a ranking of genes from higher to lower correlation with SMG1 by Pearson analysis. The obtained ranks were matched to all the pathways included in the MSigDB dataset using GSEA. The cross-presentation and TCR-signaling pathways identified through the GSEA analyses indicated that they were inversely correlated with SMG1 expression in most tumor types; confirming the importance of SMG1 values in tumor immunity, thus supporting the scRNAseq analysis and the mouse models (Fig. 1 and 2). Importantly, the pathway of IL6ST was directly correlated with SMG1 expression in most tumor types (Fig. S10J-K) in line with previous observations and indicating that IL-6 axis could be an inducer of SMG1.

We wanted to determine that SMG1 protein was induced in the presence of IL-6, and to that end we choose the B16/F10 cell line that express intermedium levels of SMG1 and low expression of IL-6. The cells were cultured in the presence of hyper-IL6 and SMG1 protein expression was determine by western blot (Fig. 7F). To further validate the relevance of IL-6/STAT3 axis in the control of SMG1 expression and thus of the NMD activity, we used different tumor-cells expressing the NMD luciferase reporter plasmid (Fig. 5B, S7C) cultured in the presence of hyper-IL6 (Fig. 7G). A reduction of luciferase signal was observed in all stable tumor cells treated with hyper-IL6, while no changes in luciferase signal was detected in any of the SMG1^KD^ stable tumor cells expressing the luciferase NMD reporter. This experiment proved that IL-6 actually induces stronger NMD activity. Then, we aimed to prove that this was also occurring in Trp53 mutated protein that leads to endogenous neoantigen (RYPAITSL) expression. 4T1 cells in culture with hyper-IL-6 showed a significant reduction in the levels of Trp53 transcript measured by qRT-PCR and no changes were detected in 4T1 SMG1^KD^ cells (Fig. 7H).

To further explore the dependence of SMG1 expression levels on the STAT3/IL-6 axis Panc02.gCtrl and STAT3^KD^ cells were treated with hyper-IL-6 for 96 h. Then, we performed a qRT-PCR to analyze SMG1 mRNA expression. A decrease in SMG1 was detected in the STAT3^KD^ cells, showing that STAT3 was involved in the upregulation of SMG1 expression in response to IL-6 stimulus (Fig. 7I).

Finally, we aimed to assess the impact of IL-6/STAT3 through disrupting immune control of antigens that are under control of the NMD checkpoint. STAT3 is a master regulator of many inflammatory and pro-tumorigenic events. For that reason, we decided to generate Panc02.STAT3^KD^ cells by CRISPR (Fig. S10L) stably transfected with the SIINFEKL-luciferase reporter plasmid. Control and STAT3^KD^ tumors selected to rendered similar luciferase signals when implanted contralaterally in the same mice (Rag2/IL2rg^-/-^) were adoptively transferred with OT-I lymphocytes (Fig. S11). Luciferase signal was tracked along the experiment. STAT3 inhibition showed a minor, but not significant impact in tumor progression despite being implanted in Rag2/IL2rg^-/-^ mice. However, upon the immune pressure elicited by the adoptive transfer of OT-I lymphocytes that recognize the SIINFEKL antigen under NMD checkpoint, we observed that STAT3^KD^ tumors were efficiently controlled, while the gCtrl tumor showed a transient inhibition of luciferase as it occurred with SMG1^KD^ tumors (Fig. 7J and K, S11).

The therapeutic effect on STAT3^KD^ tumors that underwent the immune pressure on an NMD-dependent antigen was similar to the one observed with SMG1^KD^ tumors, suggesting that both pathways are intertwined. To further address the dependencies of IL-6 on NMD in the overall outcome of tumor immunity triggered by anti-CTLA-4 and anti-PD-1 antibodies, we performed an *in vivo* experiment in 4T1 tumor-bearing mice combining IL-6 blocking antibody with anti-CTLA-4 and anti-PD-1 antibodies in control tumors or SMG1^KD^ derived tumors. In these experiments, we used a suboptimal dose of anti-CTLA4 and anti-PD-1 antibody so we might measure the additive effect of all therapeutic factors. Improvement of ICB treatment in the context of IL-6 blockade [18–20, 28] and the detrimental effect of IL-6 in response to ICB has previously been described [29] , as well as the additive effect of SMG1 inhibition with ICB treatment (Fig. S3I). There was no therapeutic improvement when both SMG1 and IL-6 were inhibited concurrently with ICB treatment; suggesting that one of the most relevant mechanisms of action for IL-6 inhibition may be related to controlling NMD activity and further confirming the here revealed cross-talk of these two pathways in determining tumor immunogenicity (Fig. 7L).

## Discussion

Herein, we experimentally prove that NMD is a process that is regulated in response to the wave of tumor inflammation limiting the efficacy of anti-cancer T cell-mediated immune responses.

Cancer immunotherapy depends on the specificity of an endogenous or adoptive transfer immune response to recognize and destroy any disseminated tumor lesion. The three main interventions in cancer immunotherapy approaches can be summarized as: A) immunotherapy strategies to reinvigorate endogenous natural immune responses aimed at counteracting immunosuppressive signals or enhancing T-cell activation, with anti-CTLA-4 and anti-PD-1 as the gold-standard and most successful treatments in oncology patients; B) adoptive cell therapy mainly aimed at engrafting an antitumor immune response, consisting of transferring tumor-infiltrating lymphocytes (TILs) or chimeric antigen-receptor T lymphocytes (CAR-T) which are the two successful approaches in solid and hematological tumors respectively; C) and tumor vaccines, ranging from generic vaccines against common cancer antigens to highly personalized vaccines (technically cumbersome but more beneficial to the patient). All cancer immunotherapy approaches, with the exception of CAR-T, rely on the presence of strong and immunodominant tumor antigens; the higher the difference of antigen with the encoded native protein, the higher the chances to mount a strong immune response. Thus, the ideal antigens are those classified as neoantigens, usually considered as cancer patient specific (private), and not expressed in any other tissue apart from the tumors. Within this group, frameshift mutations are presumably the most immunogenic ones since all amino acid sequence changes downstream the mutational site [6]. Besides, there is another type of potential tumors antigens recently described as of value [7, 30] derived from mRNA maturation errors (e.g., intron inclusion, non-canonical exon splicing, etc.) which can lead to potential frameshift errors, and thereby derived in byproduct immunogenic proteins. The fact is that both of these antigen entities derived from frameshift errors will lead to, in most cases PTC, linking its expression to NMD activity, which indicates the importance of this pathway for predicting and designing most of the current immunotherapy approaches. Thus, NMD functions as a neoantigen immune checkpoint.

As it happens with other immune related pathways (e.g., TGF-B, CTLA-4, etc.) [31, 32] the role of NMD in cancer is complex, and sometimes with apparently controversial outcomes [33, 34]. It is possible that NMD disruption in early oncogenesis creates an inflammatory and sustained environment that favors tumorigenesis [35, 36]. However, in established tumors NMD inhibition was shown to enhance tumor immunity [15–17]. Over the last few years, there has been much evidence indicating that NMD is a pleiotropic pathway [37]. NMD was claimed to play a protective tumorigenic role on tumors harboring mutation that trigger a PTC and generate truncate dominant oncogenic protein [38], in which case it would be favored lower NMD activity in tumors. In addition, supporting the protective activity of NMD in cancer is the fact that haplo-insufficient SMG1^+/-^ mice display a mildly increased of tumor incidence in older mice. It is important to highlight that the SMG1^+/-^ mice develop an auto-inflammatory phenotype in many tissues, and the persistence of this chronic multi-organ inflammation might be the cause of higher rates of cancer in the long term. More intriguing is the fact that a mutation in UPF1 (NMD factor) has been reported as a driver mutation in two different types of cancers: Inflammatory Myofibroblastic Tumors (benign tumor) [36] and in Pancreatic Adenosquamous Carcinoma [39]. Inflammatory Myofibroblastic Tumors are precisely characterized for high tumor immune infiltration and a good prognosis. On the other hand, we should not disregard that the reported UPF1 mutation in Pancreatic Adenosquamous Carcinoma might not lead to loss of NMD activity as shown by a recent study [40].

Contrary to this school of thought is the evidence indicating that low NMD levels are associated with better prognosis in many types of tumors [12, 41, 42]. In a pan-cancer study, about a fifth of all tumors showed NMD dependencies [43]. MSI tumors characterized by large mutational loads display high NMD activity and NMD function is required for tumor progression and survival [44]. Previous publications have underscored PTC containing antigens that are not eliminated by NMD impact on the response to immune-checkpoint blockade therapies [11, 45, 46]. The current work indicates that NMD activity is actually highjacked by the tumor. It also brings an important advance in the understanding of the immunosuppression cross-talk signals that operate in the tumor milieu; indicating that they do not only influence counteracting the effector function of immune cells that infiltrate the tumor, but also influence limiting the source of neoantigens derived from potential frameshift mutations that are under NMD control. This is the first evidence, to the best of our knowledge, that an intrinsic immunosuppressive pathway (IL-6/STAT3) can directly affect tumor antigenicity leading to changes in the T-cell lymphocyte repertoire and restrict antigen dependent tumor immunity. This result has potential direct implications in the response to ICB therapy, as well as on the design of personalized neoantigen vaccines that contain frameshift-derived mutations and in adoptive T-cell therapy using TILs [47].

The actual source of IL-6 might come from multiple type of cells in the TME. Endothelial cells, myeloid cells and B cells represent probably the main sources of IL-6 within the TME [48]. It has been reported that activated T cells can produce IL-6 in lower levels. However, T cell can trigger the production of IL-6 via crosstalk with other cell types within the TME through various signaling pathways (e.g., TNF-α, IL-1 or TGF-β) [49, 50]. Previous studies have also demonstrated that ICB therapy increase IL-6 levels in cancer patients, what has been associated with worse prognosis [18, 51].

NMD loss has been associated with autoimmunity events by increasing immune infiltrates and cytokine production [52]. This autoinflammatory phenomenon might be explained by the expression of aberrant antigenic protein derived from endogenous transcript generated during mRNA maturation/splicing or from other inflammatory signals induced by cell stress [7, 52, 53]. On the basis of our results we can conclude that frameshift-derived antigens controlled by NMD is a main driver in determining the fate of the immune response in the context of cancer immunotherapy. We cannot withdraw the existence of other immune factors triggered by NMD inhibition that contribute to the enhancement of the immune response when NMD is inhibited, but they seem to be less relevant than the antigenic force.

We observed that counteracting STAT3 or IL-6 signaling (Fig. 7L) overlaps to a great extent with the therapeutic effect of SMG1 inhibition, opening the possibility of developing an indirect therapeutic intervention of NMD activity by blocking IL-6. The signaling transducer of IL-6 (IL6ST) converges on STAT3 activation with some other cytokines, also underscored as pro-tumorigenic and immunosuppressive mediators (Leukemia inhibitory factor, Oncostatin M, Ciliary neurotrophic factor, Cardiotrophin-1, IL-11 and Cardiotrophin-like cytokine/novel neurotrophin-1) [54, 55]. It is plausible that most of those cytokines, as well as some other receptor signals that trigger STAT3 activation, lead to upregulation of SMG1 and therefore NMD, not to mention the tumors that acquire mutations with the constitutive active form of STAT3 [56]. Thus, depending on the type of tumor or the context that dominates the induction of STAT3, the soluble cytokine or receptor to target might vary. Obviously, pharmacological inhibition of the intracellular factor STAT3 or SMG1 will be the best approach but there are still some challenges to address [33, 34, 57, 58]. Tumor-targeting RNAi oligonucleotide therapy have been proposed as a possible therapeutic intervention and, as a matter of fact, a clinical trial to inhibit STAT3 with oligonucleotides in combination with radiation therapy is currently in phase I for Relapsed/Refractory B-Cell NHL [59]; (ClinicalTrials.gov NCT04995536). Given the stronger multifactorial pro-tumorigenic role of STAT3, it is likely to be the most desirable target to be drugged, as it orchestrates many immunosuppressive and pro-tumorigenic pathways in addition to modulating NMD function [60]. However, we should bear in mind that SMG1 inhibition shows a more profound effect than STAT3 inhibition in the tumors that express immunodominant antigen upstream a PTC (Fig. 5C vs 7I). There are also some drugs previously described that seem to inhibit NMD function indirectly and that need to be explored [33, 34]. Some of them have shown to even stabilize neoantigens derived from frameshift mutation with PTC [14]. Many of these drugs are exerting their function on other pathways in the cell and are even considered as chemotherapy drugs. Therefore, it may be difficult to dissect the actual effect of NMD inhibition in the final antitumor response. SMG1 is considered to be the main kinase that initiates the activation of NMD; however, a recent study indicates that the oncogenic AKT kinase can supplant the action of SMG1 [61, 62] complicating the therapeutic landscape and indicating that, depending on tumor type, the inhibition might be better applied on other upstream factors of the NMD pathway such as UPF1 or UPF2. Drugability of this newly discovered immune evasion pathways may be envisioned neutralizing STAT-3 activating cytokines or promoting the degradation of NMD factors with novel tools for selective targeted protein degradation [63] or mRNA [17].

## Methods

### Mice strains

C57BL/6 and Balb/c mice were purchased from Envigo. All experiments were performed using 6-8 weeks old female mice. OT-I and Rag2/IL2rg^-/-^ transgenic mice were bred in house.

### Cell lines and culture conditions

Panc02 cell line was a kind gift from Dr I Melero (CIMA, Pamplona, Spain). 4T1 cells were provided by Dr F Lecanda (CIMA, Pamplona, Spain) and B16/F10 by S. Hervás-Stubbs (CIMA, Pamplona, Spain). Cell lines were cultured in RPMI-1640 medium (4T1), Dulbecco’s modified Eagle’s medium (DMEM) (B16/10 and Panc02) (all from Gibco) supplemented with 8-10% heat-inactivated FCS, 100 U/ml penicillin, and 100 μg/ml penicillin/streptomycin. Medium for splenocytes or lymph node cells consisted in RPMI that was additionally supplemented with 1 mM sodium pyruvate (all from Gibco), 0.05 mM β-mercaptoethanol (Sigma), 1mM HEPES and 1X minimal essential medium (MEM) non-essential amino acids (all from Gibco). All cell lines and assay cultures were maintained at 37 °C and 5% CO_2_. All cells were mycoplasma-free and tested regularly using *MycoAlert*™ PLUS Mycoplasma Detection Kit (Lonza).

### CRISPR cell line generation

sgRNA CRISPR guides (gCtrl: Control sgRNA: GCGAGGTATTCGGCTCCGCG [64]; SMG1: CAAAGGCACGATGATACCAG; UPF1: GGTATTACAGTAAACCACGC; UPF2: CAGCAAACACTAATCGTGAG and STAT3: GAGATTATGAAACACCAACG and TTCGAAGGTTGTGCTGATAG) were purchased from Sigma and cloned in house in the pX458-GFP vector (Addgene plasmid #48138). The plasmids were transiently transfected in 4T1, B16 or Panc02 murine cancer cells using Lipofectamine 2000 (Invitrogen) following manufacturer instructions. The next day GFP+ cells were sorted in a MoFlo Astrios EQs (Beckman Coulter) cell sorter. CRISPR efficiency in the sorted pools was corroborated by SANGER sequencing and Tracking of Indels by Decomposition (TIDE): http://shinyapps.datacurators.nl/tide/. In addition, to generate B16.SMG1^KD^ clone, an extra sorting was performed: B16.SMG1^KD^ pool cell line was seeded in flat-bottom 96-well plate (1 cell per well). Clones obtained this way were again SANGER sequenced and TIDE checked. Western Blot was also performed to assess the protein knockdowns when an antibody was available: SMG1 (clone Q25; Cell Signaling), UPF1 (Polyclonal; Sigma) and STAT3 (clone 124H6; Cell Signaling). NMD functionality disruption was detected using pNMD^+^ reporter plasmid [65]. To explore and discard the presence of potential off-targets we first found the potential candidates using Off-Spoter (Jefferson Computational Medicine Center: https://cm.jefferson.edu/Off-Spotter/). We chose the top 2-3 candidates (attending to guide similarity) for each guide and sequenced the potential off-target hit flanking zone by SANGER to check that no CRISPR editing took place. See primers in the resources table. We also checked that SMG1^KD^ cells did not show any alterations in antigen presentation or MHC-I levels. In order to achieve this goal, we plated Panc02 and 3 x10^5^ 4T1.gCtrl or SMG1^KD^ cells in 6-well plates and incubated with IFN-γ 10^3^ U/ml or vehicle o/n. After the incubation, cells were tripsinized, washed and stained for flow cytometry with anti-mouse anti-H-2Kb/H2Db-PE (clone 28-8-6) (BioLegend) for Panc02, and anti-H-2Kd-APC (clone SF1-1.1) (BioLegend) for 4T1 cells. Data was acquired in a CytoFLEX LS flow cytometer (Beckman Coulter) and analyzed in FlowJo 10. We also coated Panc02 cells (gCtrl or SMG1^KD^) with serial dilutions of SIINFEKL peptide, washed the cells and co-cultured them in a 1:10 ratio (5 x 10^5^ cells vs. 5 x10^4^ lymphocytes) in a 96-well plate (BD) with OT-I naïve splenocytes to measure IFN-γ by ELISA (BD).

### NMD SIINFEKL-luciferase Sleeping Beauty-based plasmid stable cell lines

In phase with the EF-1α promoter pSBbi-Pur (Addgene plasmid #60523), we designed a NMD reporter cassette with the following elements: the firefly luciferase protein with a PEST motive in the 5’ end to compromise the stability of the protein and accelerate its turnover so we could detect luciferase fluctuations due to NMD activity modulation with better sensitivity. Downstream the luciferase, we inserted the coding sequence of the SIINFEKL peptide in addition to another one MHC-II-dependent flanked by 2 cathepsin sites: DDCWFYFTYSVNGYNNEAIVHVVETPDCP [26]. Finally, we inserted the β-Globin-PTC39 cassette. See plasmid scheme in Fig. 5B. Final construction was ordered to Genscript. To address whether NMD is not an absolute process, Panc02.gCtrl and Panc02.SMG1^KD^ cells were transfected with WT or mutated PTC-free version of the plasmid and were co-cultured with OT-I splenocytes in a 1:10 (Cell:Splenocytes ratio) to measure IFN-γ by ELISPOT. Assay was read o/n and spots were counted in an ImmunoSpot® device. Stable lines Panc02, B16 or 4T1 cells were plated in a 6-well plate and co-transfected with 500 μg of the reporter plasmid and 500 μg of SB100X transposase using Lipofectamine 2000 (Invitrogen). Prior to the *in vivo* studies with the Panc02.gCtrl and SMG1^KD^ cell lines, we checked for SIINFEKL presentation and chosen for similar expression in both cell lines.

### Tumor models

#### Tumor growth studies with CRISPR-edited cells

6-8-week-old female C57/BL6 mice were inoculated with 1.5 × 10^5^ B16/F10 melanoma model or 1.5 × 10^5^ Panc02 pancreas adenocarcinoma cell line. 5 × 10^4^ 4T1 breast carcinoma cells were injected in 6-8-week-old female Balb/c or Rag2/IL2rg^-/-^ immunodeficent mice (gCtrl or NMD factor knockdown as shown in legends). Tumor volumes were measured over time.

#### NMD combined with anti-CTLA-4 + anti-PD-1 ICB therapy

5 × 10^4^ 4T1 breast carcinoma cells were injected in 6-8 week-old female Balb/c. In this experiment we used a suboptimal dose of anti-CTLA-4 and anti-PD-1:100 μg of each antibody, anti-CTLA-4 (clone 9H10) anti-PD-1 (clone rmp1-14). 200 μg were injected or each antibody on days: 6, 13, 17 and 20.

#### NMD combined with anti-CTLA-4 + anti-PD-1 ICB therapy and anti-IL-6 blockade in 4T1 breast cancer model

5 × 10^4^ 4T1 breast carcinoma cells were injected in 6-8 week-old female Balb/c. In this experiment we used a suboptimal dose of anti-CTLA-4 and anti-PD-1, 100 μg of each antibody, anti-CTLA-4 (clone 9H10) anti-PD-1 (clone rmp1-14) and when indicated supplemented with 200 μg of anti-IL-6 (clone MP5-20F3) (all from BioXcell) were injected intraperitoneally (IP) at days +1, +4, +7 and +15. Control group was treated with 200 μg of Rat IgG2a isotype control (clone 2A3) (BioXcell) at the same time points.

#### Immune depletion studies

Balb/c mice were inoculated with 5 x 10^4^ 4T1.gCtrl in the right flank on day 0. Mice were treated intraperitoneally with 200 μg of anti-CD8a (clone 53-6.7) or anti-CD4 (clone GK1.5) (both from BioXcell) antibodies or anti-Asialo-GM1 (Poly21460) (BioLegend) as described previously [66] on days -4, +3, +6 and +13. Anti-IFNAR (Clone MAR1-5A3) antibody was administered intraperitoneally on days 0, +3, +6 and +13. In order to check immune depletion, a mouse per group was bled on day 23. 100 μl of blood was lysated twice using 2 ml of ACK lysis buffer (Gibco). Cells were stained with anti-mouse anti-CD8a-APC-Fire750 (clone 53-6.7), anti-CD4-BV510 (clone GK1.5) and anti-NKp46-BV605 (clone 29A1.4) (all from BioLegend) for 20 min RT protected from light at room temperature. Samples were acquired in a CytoFLEX LS flow cytometer (Beckman Coulter) and analyzed with FlowJo 10 (FlowJo). On day 23 animals were also sacrificed tumors weighted.

Tumor vaccination experiments: 4T1 VAX: 6-8-week-old female Balb/c mice were subcutaneously immunized with 5 × 10^5^ 4T1.gCtrl or SMG1^KD^ irradiated cells per leg (1 × 10^6^ total cells) in the inguinal area on days -3, 0 and +3. On day 0, 5 × 10^4^ 4T1.gCtrl cells were subcutaneously injected in the right flank of the mice. Tumor size was measured over time.

Tumor volume in all the experiments was measured using a caliper and calculated using the following formula: tumor volume = (length × width^2^)/2.

#### TP53 RYPAITSL ELISPOT

4T1.gCtrl or SMG1^KD^ tumors were established as described previously in this methods. On day 14, after the injection of the cells, mice were sacrificed and draining lymph nodes were isolated. Lymph nodes were homogenized and filtered through a 40 μm nylon cell strainer (Falcon) to a 50-mL centrifuge conical tube (Corning). Cells were spun down and diluted in lymph node cells medium (see cell culture section). To check the presence of specific T cells for the Trp53-derived peptide, an IFN-γ ELISPOT (BD) assay was performed: 10^6^ lymphocytes from the control or SMG1^KD^ group were cultured o/n in the presence of the Trp53-derived peptide (RYPAITSL) at final concentration of 10 μg/ml. The next day the play was read following manufacturer instructions. Spots were quantified as described previously.

### NMD regulation *in vivo* studies

#### Immunodeficient model

Rag2/IL2rg^-/-^ immunodeficient mice were subcutaneously injected with Panc02 pancreas cancer model stably expressing our luciferase-SIINFEKL NMD reporter plasmid. 1 x 10^6^ Panc02.gCtrl in the right flank and 1 x 10^6^ of Panc02.SMG1^KD^ in the left one. Luciferase signal was measured in a PhotonIMAGER device (Biospace Lab) on the days shown and quantified with IMARIS software (Biospace Lab). OT-I adoptive cell therapy: OT-I splenocytes were activated o/n the day before administration with 5 μg/ml LPS (Sigma) and 2 μM of OVA 257-264 peptide (SIINFEKL) (GenCust) in RPMI medium supplemented as described in the cell culture section. On day 7 of the experiment, 10^7^ OT-I cells were washed twice and injected intravenously. Rechallenge with 50 μg of SIINFEKL was administered IP on day 15.

#### PTC-free in immunodeficient model

Same protocol was reproduced as described for immunodeficient model. In this case we used cells that were transduced with a mutated variant of our luciferase-SIINFEKL NMD reporter plasmid that lacked the PTC of the β-Globin cassette.

#### Immunocompetent model

Tumors were injected as in immunodeficient mice study: 6-8-week-old female C57/BL6 mice were subcutaneously injected with 1 x 10^6^ Panc02.gCtrl in the right flank and the same number of SMG1^KD^ cells in the left one. 100 μg anti-mouse anti-CTLA-4 and anti-PD-1 were IP-administered on days +1, +4 and +7. Luciferase signal was measured in a PhotonIMAGER on the days shown.

### SMG1 regulation by IL-6/STAT3 axis

#### Cytokine measurement by flow cytometry

Rag2/IL2rg^-/-^ immunodeficient mice or C57/BL6 mice were injected with Panc02 pancreas cancer model stably expressing our luciferase-SIINFEKL NMD reporter plasmid. 1 x 10^6^ Panc02.gCtrl in the right flank and 1 x 10^6^ of Panc02.SMG1^KD^ in the left one or tumor-free as indicated in the figure legends. Treatment schedule was followed as in the luciferase NMD regulation *in vivo* studies. Rag2/IL2rg^-/-^ mice were bled on day 10 and C57/BL6 on day 14. Blood was clotted during 2 h at room temperature, centrifuged 15 minutes at 1,000 g and supernatant was collected for cytokine analysis. 50 μl of sera samples were stained using BD Cytometric Bead Array (CBA) Mouse Inflammation Kit (BD) and analyzed in a CytoFLEX LS flow cytometer (Beckman Coulter).

#### In vitro

4.5 x 10^4^ B16, 10^5^ 4T1 and 3 x 10^4^ Panc02.gCtrl or SMG1^KD^ were seeded in a 6-well plate. For B16.SMG1^KD^ we used our 100% inhibited clone. All cell lines express our WT version of the luciferase-SIINFEKL NMD reporter plasmid. Cell lines were cultured in presence of 20 ng/ml recombinant mouse IL-6/IL-6Rα Protein Chimera (hyper-IL-6) or vehicle for 96 h. Luciferase signal was measured using ONE-Glo™ luciferase Assay System (Promega).

#### STAT3 *in vivo*

To address if we were able to disrupt the mechanism of NMD regulation by the IL-6/STAT3 axis, we injected Rag2/IL2rg^-/-^ immunodeficient mice with 1 x 10^6^ Panc02.gCtrl in the right flank and 1 x 10^6^ Panc02.STAT3^KD^ in the left one. We followed the same schedule for OT-I activation and SIINFEKL administration as in the first immunodeficient model experiment (see NMD regulation studies section of this Materials and methods).

### Tumor infiltrate studies by flow cytometry

#### Tumor samples

Tumors were resected on day 14 after implantation. Tumors were placed each in 100/15 mm Petri dishes (Greiner Bio-One) and digested with 5ml of medium containing collagenase D and DNase I (Both from Roche) 30 min at 37°C. After incubation, 100 μl of EDTA (Invitrogen) were added to tumors in order to stop the reaction. Samples were homogenized and filtered through a 40-μm nylon cell strainer (Falcon) to a 50-ml centrifuge conical tube (Corning). Cells were pelleted at 1,700 rpm for 5 min RT. Supernatants were discarded, and erythrocytes were lysated using 1 ml of ACK lysis buffer (Gibco) for 1 min on agitation. PBS buffer containing EDTA 2mM (Gibco); BSA (Sigma) 5mg/ml was added up to 50 mL to neutralize the lysis, and cells were spun down again at 1,700 rpm for 5 min RT. The pellet was re-suspended in 200 μl of the same PBS buffer and were spun down in a V-bottom 96-well plate (ThermoFisher) at 1,700 rpm 1 min RT. Cells were resuspended in 80 μl of Zombie UV (BioLegend) diluted 1:200 in protein-free PBS-EDTA buffer and incubated for 15 min RT protected from light. Antibody mix: CD45-PerCP-Cy5; CD8a-APC-Fire750; CD4-BV510; CD25-APC (all from BioLegend) and FOXP3-PE (Invitrogen) was added next (20 μl per sample) without washing and incubated for 20 min RT in protected from light. After this stage, cells were washed twice and fixed with Cyotofix/Cytoperm buffer (BD) for 10 min at 4°C protected from light. Samples were washed twice again to discard PFA rests and acquired using a CytoFLEX LS flow cytometer (Beckman Coulter). Data was analyzed using FlowJo 10 (FlowJo). For FOXP3 staining after the surface antibody markers cells were permeabilized using eBioscience FOXP3/transcription factor buffer set (eBiosciences) following manufacturer instructions. See gating strategy in Fig. S5A-B.

### Western Blot

Tumor cells were homogenized in lysis buffer: PBS containing 10% Triton X-100 (Sigma) with cOmplete™ Protease Inhibitor Cocktail (Roche) for 30 min in ice. Samples were then centrifuged for 15 min at 10,000 rpm 4°C. Protein concentration in the resulting supernatants was quantified using Protein Assay Dye Reagent Concentrate (BioRad) diluted in deionized water. Equal amounts of lysates were fractionated by BioRad mini-PROTEAN TGX 4-15% gels (BioRad) for SMG1 WB or 10% SDS-PAGE for UPF1 and STAT3 and electrotransferred to 0.45μm pore size nitrocellulose membranes (BioRad). After blocking with TBS (BioRad)/0.1% Tween (Sigma)-20/5% milk, the membranes were probed with rabbit anti-mouse SMG1 (Cell Signaling; 1:1,000; clone Q25), rabbit anti-mouse UPF1 (Sigma; 1:200; Polyclonal), mouse anti-mouse STAT3 (Cell Signaling; 1:1,000; clone 124H6), and rabbit anti-mouse β-Actin (Cell Signaling; 1:2,000; clone 13E5) o/n in agitation at 4°C. HRP-linked anti-rabbit or anti-mouse antibody (both from Cell Signaling; 1:5,000) were used as secondary antibodies. Protein bands were detected by chemoluminiscence using Amersham™ ECL™ Western Blotting Detection Reagents or Amersham™ ECL™ Prime Western Blotting Detection Reagents (GE Healthcare) in a ChemiDoc device (BioRad).

### TCRseq

Panc02.gCtrl or SMG1^KD^ tumors were established as previously described. Similar experiment was performed also for 4T1.gCtrl and SMG1^KD^ tumors in Balb/c mice. Tumors were subcutaneously injected in the right flank of the animals and on day 14, after the implantation of the cells, mice were sacrificed and draining lymph nodes were isolated to extract genomic DNA (gDNA).

1500 ng of gDNA from cells obtained from the draining lymph nodes were used for TCR sequencing with the Oncomine™ mouse TCR Beta-SR DNA Assay (Thermo Fisher Scientific) according to the manufacturer's instructions. Library construction and sequencing (IonTorrent S5) was performed in the Genomics Facility at CIMA Lab Diagnostics (Spain, Pamplona).

Bioinformatics analysis of the data was performed using MiXCR software filtering the top 50 clones from productive TCRB per sample follow with Immunarch R package (10.5281/zenodo.3367200).

### RNAseq

RNA sequencing was performed by adapting the technology of SCRB-Seq [67] to allow for the high cost-efficient multiplexed transcriptome characterization. Briefly, poly- (A)+ RNA was purified using the Dynabeads™ mRNA DIRECT™ Purification Kit (ThermoFisher Scientific). poly- (A)+ RNA was annealed to a custom primer containing a poly- (T) tract, a Unique Molecule Identifier (UMI), and a sample barcode. Retrotranscription using Template-switching oligonucleotides (TSO) was then used to synthetize and amplify 3’UTR enriched cDNA, resulting in barcoded cDNA fragments. Library preparation was performed using the Nextera XT library preparation protocol which introduces i5-P5 and i7-P7 structure for massive parallel sequencing. Quality control was performed following pre-amplification RT and library preparation to ensure quality and length accuracy, as well as to equilibrate sample pooling. Libraries were then circularized and sequenced using a DNBSeq-G400 sequencer (MGI), using the MGIEasy Circularization Kit (MGI). Approx. 30 million pair-end reads (2x100 bp) were sequenced for each sample. Raw sequences were called using Zebra caller (MGI) and demultiplexed using Cutadapt. RNAseq was carried out at the Genomics Unit of the Center for Applied Medical Research (CIMA, Universidad de Navarra).

Sequencing reads were trimmed with Trimmomatic (LEADING:3, TRAILING:3, SLIDINGWINDOW:4:15, MINLEN:36) and aligned to GRCm38.p6 (release 101, genome-build-accession NCBI:GCA_000001635.8) with STAR using two-pass-mode. Variant calling was performed following the GATK best practices workflow. Precisely duplicates were marked with Picard and base recalibrated based in know variants (dbSNP_150, including SNPs and indels). Haplotypes were called and hard filter (snvs: "QD < 2.0 || FS > 60.0 || MQ < 40.0 || MQRankSum < -12.5 || ReadPosRankSum < -8.0"; indels: "QD < 2.0 || FS > 200.0 || ReadPosRankSum < -20.0”). Finally, variation effect was annotated with VEP. They were converted into a customized FASTA database. R scripts and the Ensembl v101 protein FASTA file as reference were used to extract an 80 aminoacid substring containing the region of each identified variant. Then, the reference aminoacids were changed to the variant aminoacids [68]. The list of proteins were run through netMHCpan software for predicting strong binding peptides to H2Kd, H2Dd or H2Ld with predicted Kd affinity <50 nM. Out of the top candidates, with chose five peptides to assess the induction of endogenous class I mediated immune response upon tumor implantation. The frameshift identified in the RNAseq was supervised using IGV software for visualization.

### scRNAseq

BRCA scRNAseq read count data was downloaded from http://biokey.lambrechtslab.org. PDAC from [23]. UAD scRNA processed data was downloaded from the NCBI Gene Expression Omnibus database (accession code GSE131907) [22]. Seurat R package was used for analysis (https://satijalab.org/seurat/). Seurat parameters were used following the criteria described by Bassez et al., 2021 for BRCA [21]. Patients samples were separated based on SMG1 expression in the tumor cells into high (> median) and low (< median). UMAP graph of each group of patient (SMG1 high and SMG1 low) was processed using the Spectre R package. TCR scRNAseq clonotype data was obtained from http://biokey.lambrechtslab.org. Patients with less than 2 common clonotypes in the pretreatment sample versus on anti-PD-1 treatment were withdrawn from the analysis. Positive expansion on TCR clones upon anti-PD-1 treatment was considered positive when the expansion increases with a p-value < 0.002 using a paired t-test. Change in SMG1 expression from pre vs. on treatment samples was determine considering the ratio of expression and intensity of SMG1 in tumor cells, the SMG1 change was graph using ggplot (https://ggplot2.tidyverse.org/index.html) and ggalt (https://CRAN.R-project.org/package=ggalt) R packages. Intensity of gene expression for SMG1, IL6ST and STAT3 in cancer cells was assess using nebulosa R package. For Pearson correlation analysis for expression of SMG1 vs IL6ST and STAT3 we used the ggpubr R package.

### Identification of STAT3 binding sites to SMG1 mining ChiP-seq datasets

The STAT3 ChIP-seq data was obtained from [27]. The bed file GSE67183 with MACS peak calling information was downloaded from GEO. The GSE67183_VS54_Th17_STAT3_binding_sites.bed and the bam files aligned from SRR1925779 to GRCh37-hg19 genome were imported for visualization using Gviz R package using plotTrack function [69]. We also used UCSC Genome Browser (https://genome.ucsc.edu/) data to corroborate our MACS peak findings, analyzing hg19/human genome build. STAT3 binding sites were obtained from the Transcription Factor ChIP-seq Clusters (161 factors) from ENCODE with Factorbook Motifs track in detected in GM12878 HeLa-S3 and MCF10A-Er-Src cell lines. Chromatin accessibility data was displayed employing H3K27Ac Mark from ENCODE track and DNase I hypersensibility with DNaseI Hypersensitivity Clusters from ENCODE (V3) track.

### Identification of SMG1 pathways dependencies using GSEA ranking, retrieve from TCGA gene expression dataset

RNAseq expression for each TCGA tumor type was downloaded using the RTCGA R package (DOI: 10.18129/B9.bioc.RTCGA), gene columns with null and low expression were filtered. Pearson correlation of SMG1 expression via comple.obs for all the genes was processed using cor function from stats in R. Pearson r values were used to rank the genes from direct to inverse correlation with SMG1. The list of ranked genes were matched to all gene-pathways encompassed in (msigdb.v7.0.symbols.gmt), using fgsea R pakage (DOI: 10.18129/B9.bioc.fgsea). The gene-pahtways NES scores and p-values obtained from the GSEA analysis were represented using ggplot2 V4 R package for each type of tumor.

### Survival analysis associated with SMG1 expression retrieve from TCGA dataset

Survival clinical data and RNAseq data from the TCGA was retrieved using RTCGA R package and survival analysis associated with SMG1 expression was processed using KmTCGA function. We choose the tumor entities with more 100 patients per cohort. The optimal cutpoint of variables was estimated using the surv_cutpoin function with a value of minprop of 0.3 processed with surviminer R package (https://CRAN.R-project.org/package=survminer).

### Statistics

Data obtained from *in vivo* and *in vitro* experiments data was analyzed using GraphPad Prism 7.0 (GraphPad) if it is not indicated otherwise, and all figures show mean ± SEM. Flow cytometry analysis were performed with FlowJo 10 (Tree Star). Error bars represent standard error of the mean (SEM) in all plots. One-way ANOVA followed by post-hoc Bonferroni test was performed to analyze statistical differences between independent groups. If experiments had only 2 groups, 2-tailed t-test was carried out instead. For *in vivo* experiments with measures distributed in several time points, significant effect was determined by using Two-way ANOVA with Bonferroni test. Statistical significance is considered at *p*<0.05 if in the legend of the figure is not indicated another criterion. When differences are statistically significant, the significance is always represented with asterisks (^∗^) according the following values: *p*<0.05 (^∗^), *p*<0.01 (^∗∗^), *p*<0.001 (^∗∗∗^) and *p*<0.0001 (^∗∗∗∗^). For *in vivo* experiments with several time points, asterisks show the significance of the final one.

## Conclusions

In summary, we showed that the IL-6/STAT3 axis mediates the upregulation of SMG1, which induces higher NMD activity limiting the expression of frameshift-derived neoantigens containing PTCs. We uncovered an unpredicted immunosuppressive function of IL-6/STAT3 pathway in cancer cells affecting the expression of potent neoantigens under the control of NMD. This is a new type of transient and regulated cancer immunoediting process that operates under the control of inflammatory cytokines (IL-6) orchestrating the induction of a reversible immune-excluded tumor microenvironment. SMG1 upregulation by IL-6/STAT3 is exacerbated in the context of cancer immunotherapy as there are higher levels of IL-6 hampering the efficacy of the treatment. Counteracting SMG1 or IL-6/STAT3 avoids the PTC-neoantigen escape, rescuing the efficacy of the immunotherapy. This study, moreover, underscores the importance of considering STAT3/SMG1 expression with regard to designing and predicting future immunotherapy developments. It highlights the possibility of using IL-6/STAT3/SMG1 pharmacological inhibitors to sensitize them for cancer immunotherapy.

## Supplementary Information

**Additional file 1.**

**Additional file 2.**

## Data Availability

The RNAseq high-throughput data was deposited in SRA BioPRoject PRJNA895222. Any additional information or material will be available upon reasonable request.

## Abbreviation

BRCA: breast cancer
CD3: Cluster of differentiation 3
CD4: Cluster of differentiation 4
CD8: Cluster of differentiation 8
CESC: Cervical squamous cell carcinoma and endocervical adenocarcinoma
CRISPR: Clustered regularly interspaced short palindromic repeats
CTLA-4: Cytotoxic T-Lymphocyte Antigen 4
EJC: Exon junction complex
ESCA: Esophageal carcinoma
FDA: US Food and Drug Administration
FOXP3: Forkhead Box P3
GBM: Glioblastoma multiforme
GSEA: Gene Set Enrichment Analysis
ICB: Immune-checkpoint blockade
IFN: Interferon
IFNAR: Interferon-alpha receptor
IL-6: Interleukin 6
IL-6R: Interleukin 6 Receptor
IL6ST: Interleukin-6 signal transducer
IP: Intraperitoneal injection
KD: Knock-down
KICH: Kidney Chromophobe
KIRC: Kidney renal clear cell carcinoma
KIRP: Kidney renal papillary cell carcinoma
LAG3: Lymphocyte Activating 3
LGG: Low-grade gliomas
LIHC: Liver hepatocellular carcinoma
LUAD: Lung Adenocarcinoma
LUSC: Lung squamous cell carcinoma
MHC-I: Major histocompatibility complex I
MHC-II: Major histocompatibility complex II
NK: Natural Killer cell
NMD: Nonsense-mediated mRNA decay
OV: Ovarian serous cystadenocarcinoma
PAAD: Pancreatic Adenocarcinoma
PCPG: Pheochromocytoma and Paraganglioma
PD-1: Programmed cell death protein 1
PDAC: Pancreatic ductal adenocarcinoma
PRAD: Prostate adenocarcinoma
PTC: Premature stop codons
SARC: Sarcoma
SKCM: Skin Cutaneous Melanoma
SMG1: Nonsense Mediated MRNA Decay Associated PI3K Related Kinase
SMG5: SMG5 Nonsense Mediated MRNA Decay Factor
SMG7: SMG7 Nonsense Mediated MRNA Decay Factor
STAT3: Signal Transducer And Activator Of Transcription 3
STES: Esophagus-stomach cancer
TCGA: The Cancer Genome Atlas
TCR: T cell receptor
TGCT: Testicular Germ Cell Tumors
TGF-β: Transforming growth factor beta
THCA: Thyroid cancer
THYM: Thymoma
TIDE: Tracking of Indels by DEcomposition
TIL: Tumor-infiltrating lymphocyte
TIM3: T Cell Immunoglobulin Mucin Family Member 3
TOX: Thymocyte Selection Associated High Mobility Group Box
TRAC: T Cell Receptor Alpha Constant
TRBC: T Cell Receptor Beta Constant
UCEC: Uterine Corpus Endometrial Carcinoma
UMAP: Uniform manifold approximation and projection
UPF1: Up-Frameshift Suppressor 1 Homolog
UPF2: Up-Frameshift Suppressor 2 Homolog
UPF3B: Up-Frameshift Suppressor 3 Homolog On Chromosome X
