# Supplementary material for: IL-6/STAT3 signaling in tumor cells restricts the expression of frameshift-derived neoantigens by SMG1 induction
Source: Mol Cancer. 2022 Nov 28;21:211. doi: 10.1186/s12943-022-01679-6 (PMC9703761; doi:10.1186/s12943-022-01679-6)
Supplement: Supplementary file 1 — Additional file 1. [file 12943_2022_1679_MOESM1_ESM.docx]

Supplementary information

Table S1. Human cancer cell lines set used in Figure S10G

| H1436 | Small cell lung cancer; Stage E |
| --- | --- |
| H187 | Lung carcinoma |
| H69 | Lung carcinoma |
| H460 | Lung carcinoma |
| H1437 | Adenocarcinoma; Non-small cell lung |
| H1568 | Adenocarcinoma; Non-small cell lung cancer |
| H1648 | Lung; Adenocarcinoma; Stage 3A |
| H1650 | Adenocarcinoma; Bronchoalveolar carcinoma; Stage 3B |
| H1975 | Adenocarcinoma; Non-small cell lung cancer |
| H2228 | Adenocarcinoma; Non-small cell lung |
| HCC44 | Adenocarcinoma; Non-small cell lung cancer |
| HCC827 | Lung; Adenocarcinoma; epithelial cell |
| LxF289 | Adenocarcinoma; Non-small cell lung cancer |
| PC14 | Adenocarcinoma; Non-small cell lung cancer |
| H1703 | Non Small Cell Lung Cancer |
| H1869 | Non Small Cell Lung Cancer; Stage 4 |
| HCC366 | Non Small Cell Lung Cancer; squamous cell |
| 531 MII | Ostesarcoma |
| T98 | Glioblastoma |
| U87 | Glioblastoma |
| COLON 20 | Colorectal tumor |
| CHLA 266 | Atypical teratoid rhabdoid tumor |
| CHLA 06 | Atypical teratoid rhabdoid tumor |
| BT12 | Atypical teratoid rhabdoid tumor |
| NTERA | Lung embryonal carcinoma |
| JHH6 | Adult hepatocellular carcinoma |
| MDA | Breast adenocarcinoma |
| MCF7 | Breast adenocarcinoma |
| MCF10 | Breast cancer |

.
