# Supplementary material for: IL-6/STAT3 signaling in tumor cells restricts the expression of frameshift-derived neoantigens by SMG1 induction
Source: Mol Cancer. 2022 Nov 28;21:211. doi: 10.1186/s12943-022-01679-6 (PMC9703761; doi:10.1186/s12943-022-01679-6)
Supplement: Supplementary file 2 — Additional file 2. [file 12943_2022_1679_MOESM2_ESM.pdf]

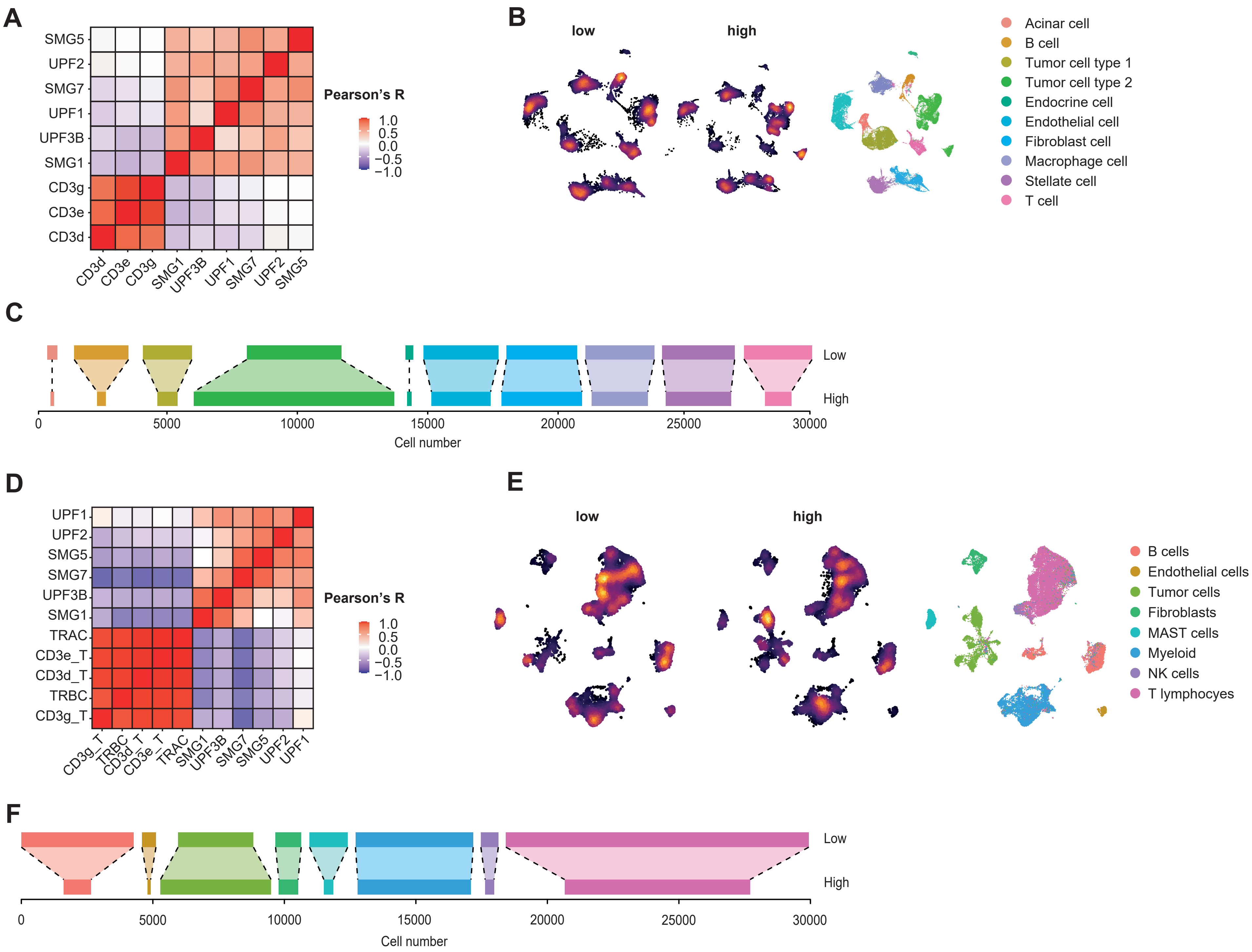

S1. SMG1 expression inversely correlates with immune infiltration in PDAC and LUAD tumors.
(A) Heatmap illustrates correlation values between the expression of genes codifying NMD factors and immune response-related genes in PDAC patients. scRNAseq data from Peng et al., 2019. B) UMAP depicting general immune populations and tumor cells in PDAC patients. Stratification in groups as low and high SMG1-expression in tumor cells. Data from Peng et al., 2019. (C) Proportion of each cell population in absolute numbers. T-cell and B-cell clusters are the most enriched in SMG1 low group. Data from (B). (D) Heatmap illustrating inverse correlation between the expression of genes codifying NMD factors and immune response-related genes in LUAD patients. scRNAseq data from Kim et al., 2020. Heatmap plots Pearson's r coefficient. (E) UMAP depicting general immune populations and tumor cells in LUAD patients. Low SMG1-expressing patients show a higher level of immune infiltration. Data from Kim et al., 2020. (F) Proportion of each cell population in absolute numbers. T-cell and B-cell clusters are the most enriched in SMG1 low group.

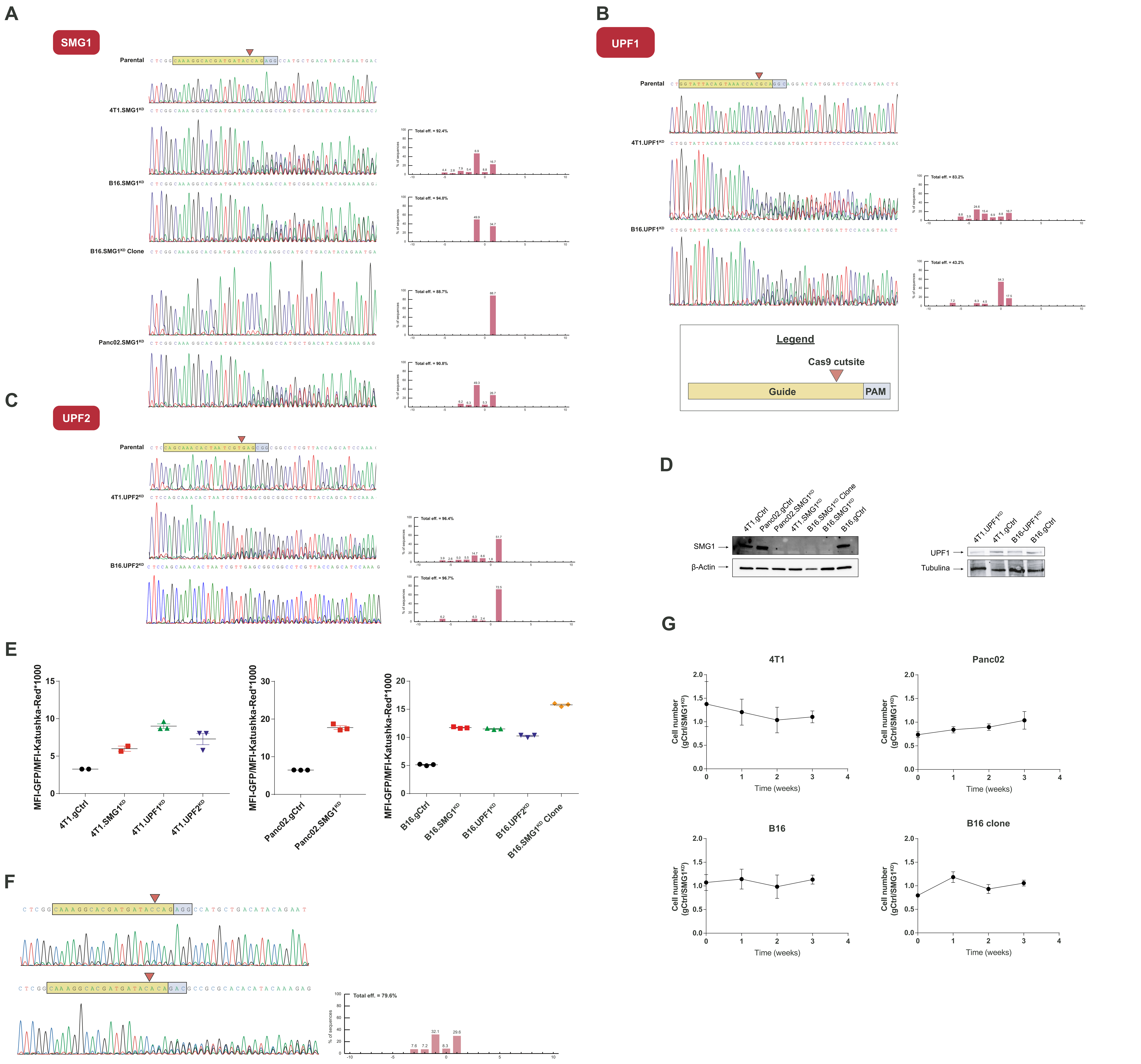

S2. SMG1, UPF1 and UPF2 CRISPR cell line validation. (A-C) Sanger sequencing and TIDE histograms showing genomic edition for the intended NMD factors. (D) WB showing protein depletion of SMG1, UPF1 and STAT3 by CRISPR. (E) Functional NMD inhibition accomplished by CRIPSR using pNMD+ reporter plasmid from Pereverzev et al., 2015. This plasmid contains GFP in phase with a PTC and Katushka-Red protein to normalize protein level. GFP increase correlates with NMD inhibition. (F) CRISPR editing is maintained over 3 months in vitro. Panc02.SMG1^{KD} shown. (G) In vitro growth of SMG1^{KD} cells compared to gCtrl measured over 3 weeks.

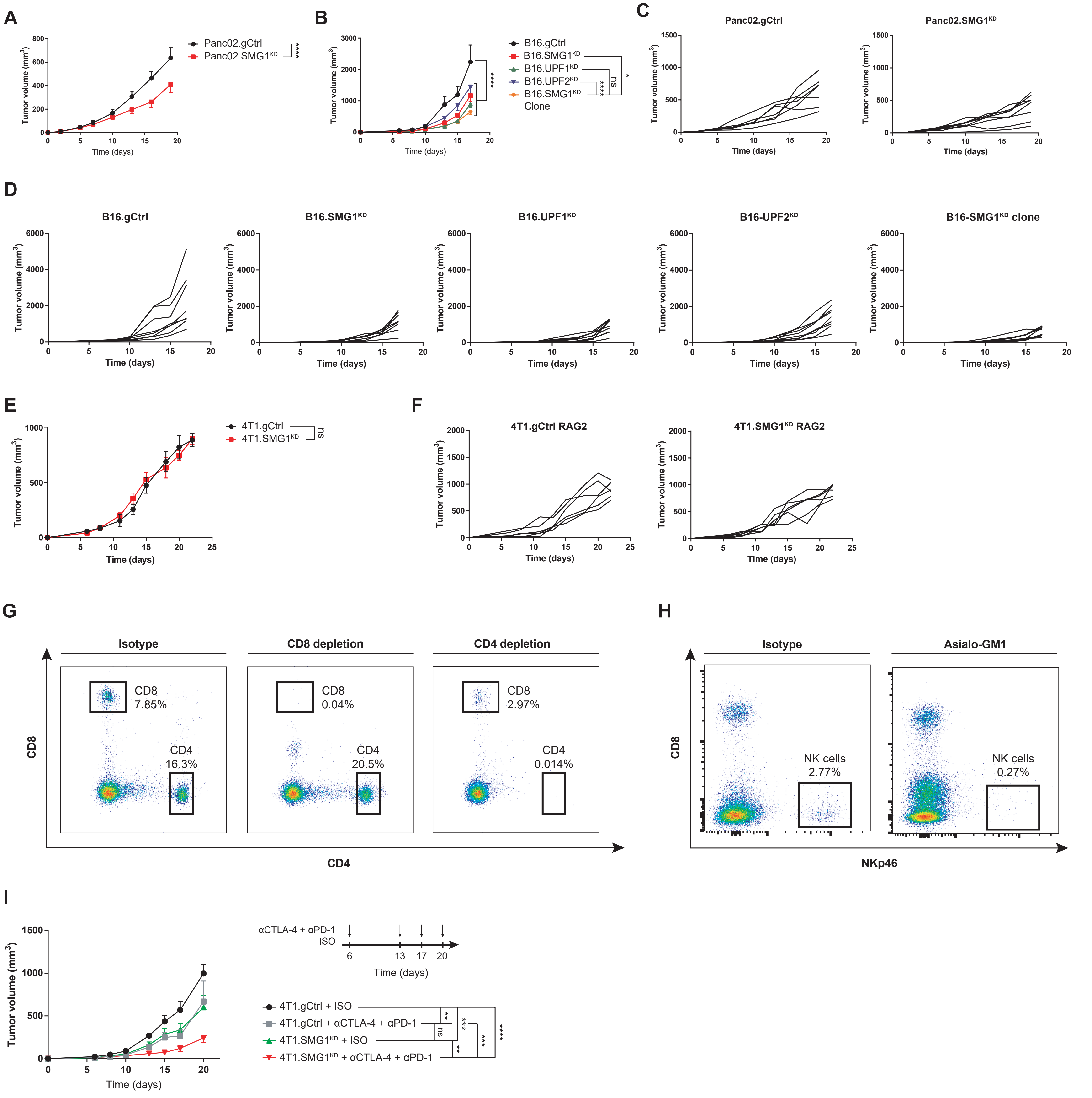

S3. NMD CRISPR inhibition enhances tumor immunity. (A) SMG1 was CRISPR knocked down in Panc02 pancreas cancer mouse model and implanted subcutaneously in C57/BL6 mice. n = 7-8/group. (B) NMD factors (SMG1, UPF1 and UPF2) were CRISPR knocked down in B16 melanoma mouse model and sorted cell pools were injected subcutaneously in C57/BL6 mice. Additionally, an absolute NMD-inhibited SMG1^{KD} B16 clone was also included. Tumor growth was measured over time. n = 8-9/group. (C) Individual tumor volumes over time from (A). (D) Individual tumor volumes over time from (B). (E) Rag2/IL2rg$^{-/-}$ mice were injected subcutaneously with 4T1.gCtrl or SMG1^{KD} cell lines. Tumor growth was measured over time. n = 6/group. (F) Individual tumor volumes of (E). (G) Isotype, CD8 and CD4 T cell depletion controls for Figure 2C. A mouse randomly selected from each group was bled. Blood was lysated and stained for flow cytometry. (H) Isotype and Asialo-GM1 control for NK depletion. Same procedure was performed as in (G). (I) Tumor volume over time of 4T1.gCtrl or SMG1^{KD} cells in Balb/c mice treated with 200 µg of isotype (clone 2A3) or 100 µg of anti-CTLA-4 (clone 9H10) and 100 µg of anti-PD-1 (clone rmp1-14) antibodies (see treatment schedule). n = 7-9/group. p-values are shown for 2-way ANOVA with Bonferroni's post-hoc test for tumor growth experiments. *p < 0.05, **p < 0.01, ***p < 0.001.

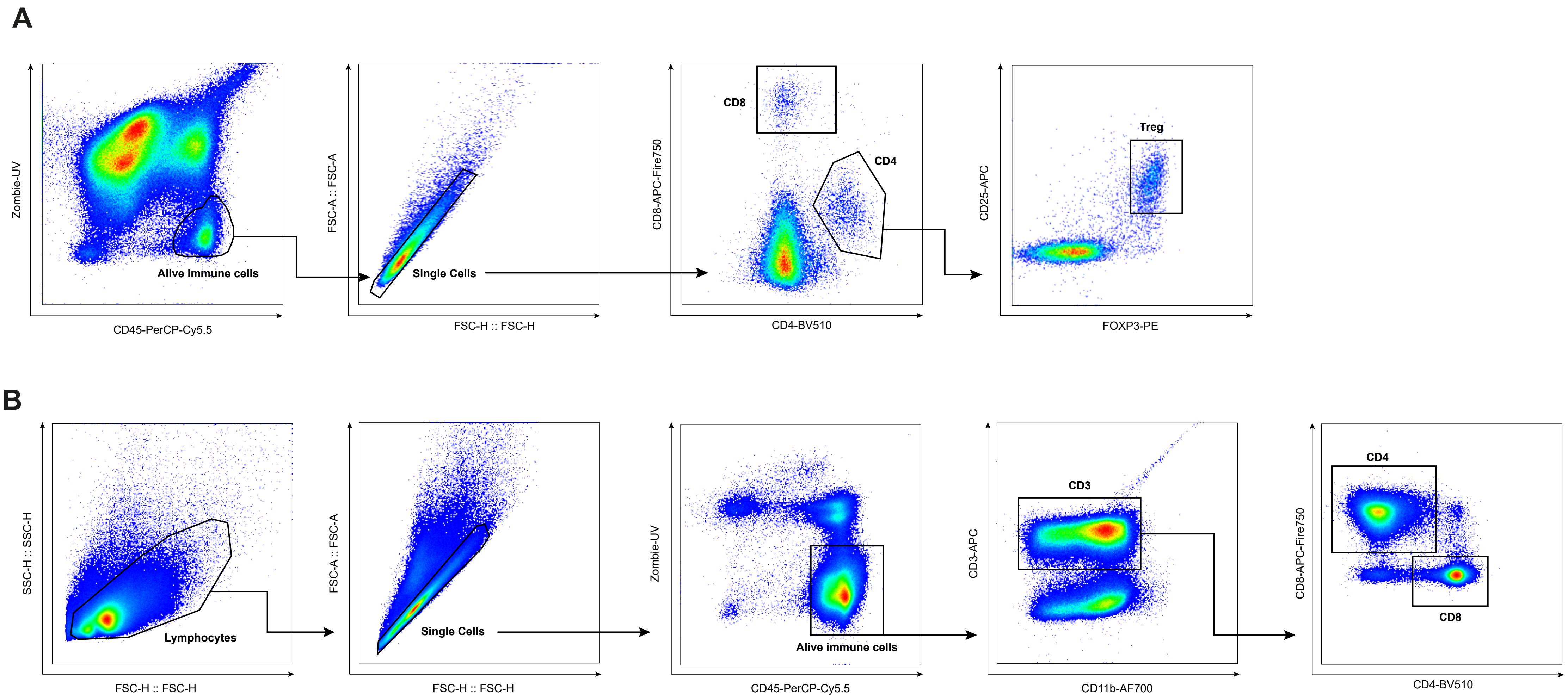

S4. Immune cell infiltration in SMG1^{KD} tumors.
(A) Gating strategy for characterization of 4T1 and Panc02 tumor infiltrate samples. Same pipeline was employed for both models. After tumor homogenization and lysis samples were stained with labelled antibodies (see Materials and Methods section). Expression data was collected by flow cytometry and analyzed using FlowJo. Statistical analysis was performed with GraphPad Prism 7.0. Immune infiltrate was identified by Zombie UV negative (live population) and CD45^{+} cells prior to selecting single cells. CD4 and CD8 populations were gated afterwards. Tregs were identified as FOXP3^{+}CD25^{+} within the CD4 T-cell subset.
(B) Gating strategy for characterization of 4T1 and Panc02 tumor infiltrate samples. Lymph nodes were homogenized and stained with antibodies (see Materials and Methods section). Data was obtained by flow cytometry and analyzed with FlowJo software. Statistical analysis was again performed with GraphPad Prism 7.0. Cells were first gated according to SSC-H and FSC-H and then single cells were selected. Live immune cells were identified as Zombie-UV^{-}CD45^{+} clusters. Total T cells were gated as CD3^{+}CD11b^{-} to discard possible false positives from myeloid population. CD4^{+} and CD8 cells were identified within the T-cell cluster.

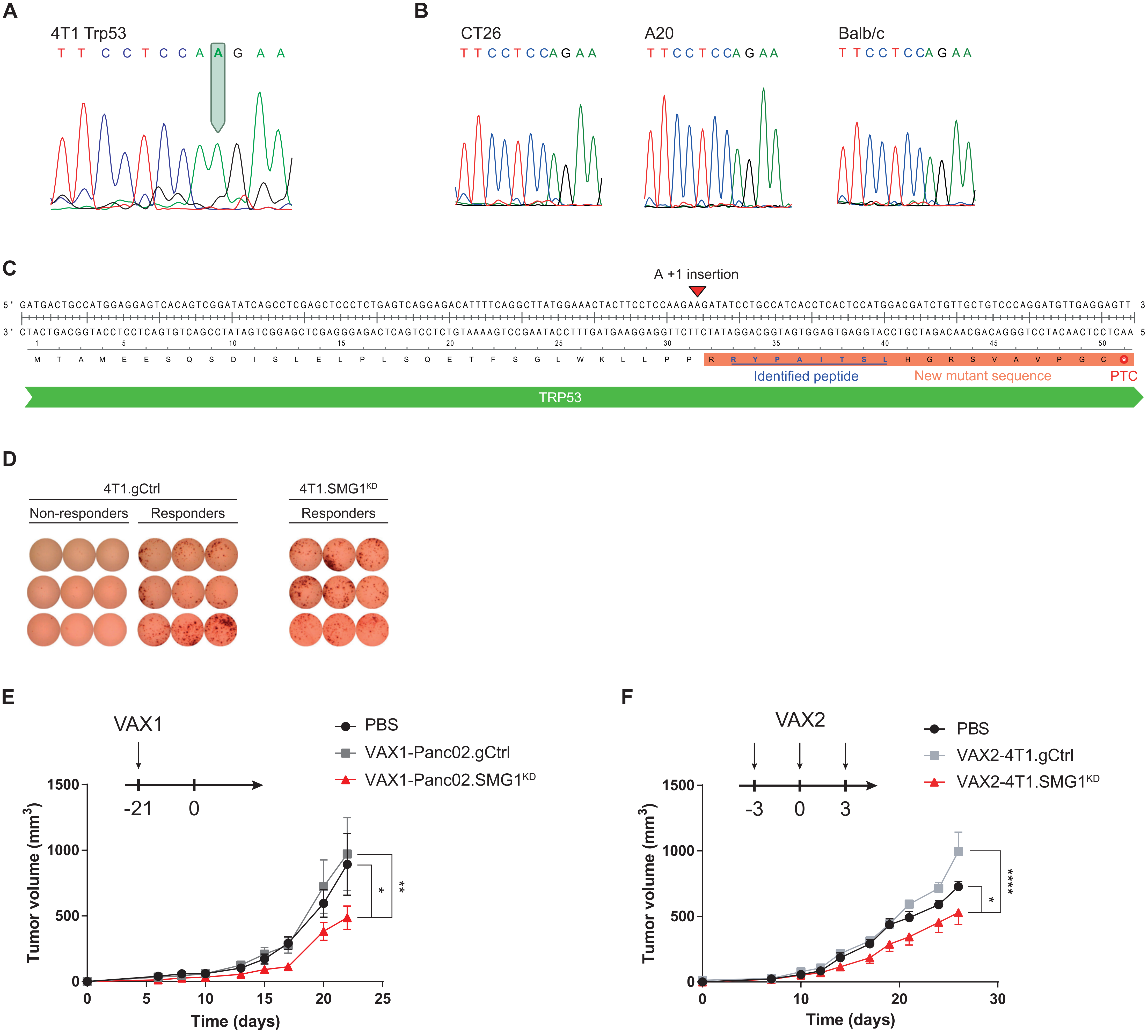

S5. SMG1 reduction improves the immune response against NMD-regulated neoantigens.(A) Sanger sequencing of Trp53 in 4T1 cDNA to confirm frameshift insertion. (B) From left to right: Trp53 Sanger sequencing of CT26 and A20 Balb/c tumor models. Sanger sequencing of cDNA of WT Balb/c extracted from the tail. (C) Genomic sequence of 4T1 cells showing the +1 insertion generating a novel aberrant sequence that serves as source of a novel neaoantigen. (D) Representative caption of the ELISPOT wells from Figure 3F. (E) 5 x 10^3 Panc02.gCtrl or $SMG1^{KD}$ were injected on -21. On day 0 tumors were subcutaneously injected. n = 6-9/group. (F) Irradiated 4T1.gCtrl or $SMG1^{KD}$ were inguinally injected on the days shown. On day 0 4T1.gCtrl tumors were established and tumor growth was measured over time. n = 6-8/group.

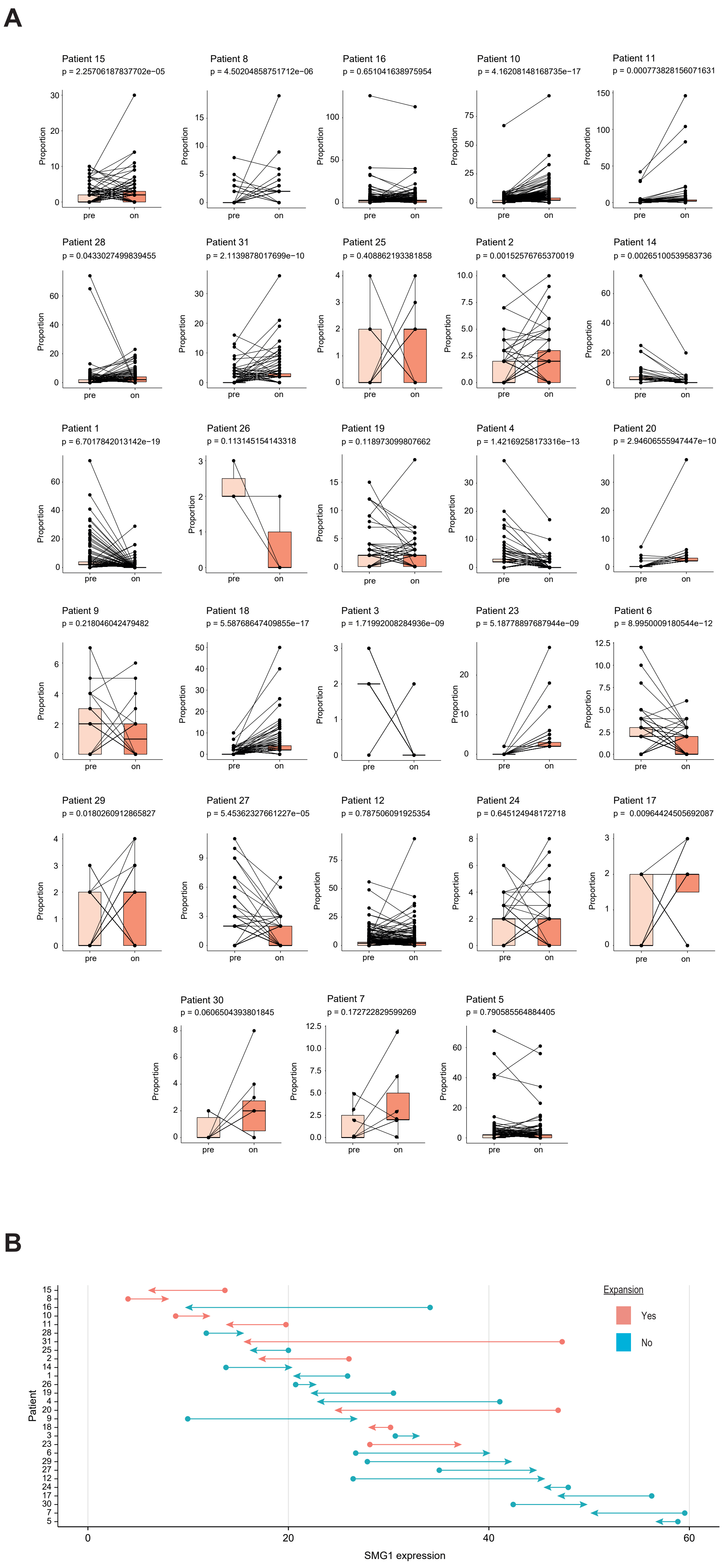
A
Patient 15
p = 2.25706187837702e-05
Patient 8
p = 4.50204858751712e-06
Patient 16
p = 0.651041638975954
Patient 10
p = 4.16208148168735e-17
Patient 11
p = 0.000773828156071631
Patient 28
p = 0.0433027499839455
Patient 31
p = 2.1139878017699e-10
Patient 25
p = 0.408862193381858
Patient 2
p = 0.00152576765370019
Patient 14
p = 0.00265100539583736
Patient 1
p = 6.7017842013142e-19
Patient 26
p = 0.113145154143318
Patient 19
p = 0.118973099807662
Patient 4
p = 1.42169258173316e-13
Patient 20
p = 2.94606555947447e-10
Patient 9
p = 0.218046042479482
Patient 18
p = 5.58768647409855e-17
Patient 3
p = 1.71992008284936e-09
Patient 23
p = 5.18778897687944e-09
Patient 6
p = 8.99500091805444e-12
Patient 29
p = 0.0180260912865827
Patient 27
p = 5.45362327661227e-05
Patient 12
p = 0.787506091925354
Patient 24
p = 0.645124948172718
Patient 17
p = 0.00964424505692087
Patient 30
p = 0.0606504393801845
Patient 7
p = 0.172722829599269
Patient 5
p = 0.790585564884405
Proportion
pre
on
B
Expansion
Yes
No
Patient
15
8
16
10
11
28
31
25
2
14
1
26
19
4
20
9
18
3
23
6
29
27
12
24
17
30
7
5
0
20
40
60
SMG1 expression

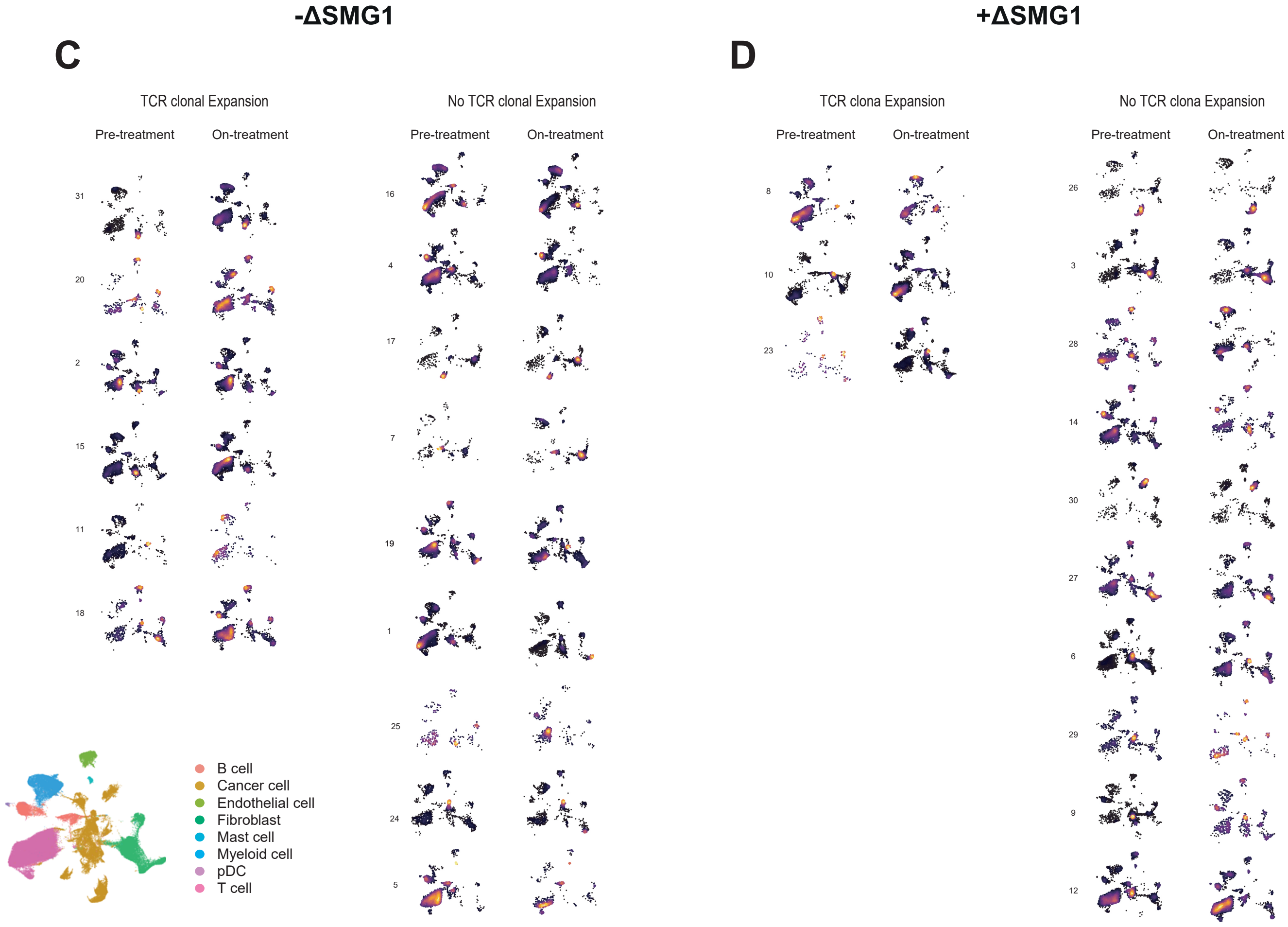

S6. SMG1 downregulation in BRCA patients on anti-PD-1 treatment triggers T-cell clonal expansion. (A) TCR clon proportions in BRCA patients before (pre) or anti-PD-1 treatment. P-value expresses significance in the number of clones detected in patients pre or on treatment. n = 28 patients. (B) Dumbbell-based graph depicting the expression of SMG1 in BRCA patients pre (dot) and on anti-PD-1 treatment (arrow). Color legend shows whether TCR clonal expansion takes place. n = 28. (C) UMAP depicting the different cell subpopulations for each patient in pre-treament and on anti-PD1 treatment for the BRCA cohort with reduction of SMG1 expression in malignant cell upon anti-PD1 treatment.
(D) UMAP depicting the different cell subpopulations for each patient in pre-treament and on anti-PD1 treatment for the BRCA cohort with increse SMG1 expression in malignant cell upon anti-PD1 treatment.

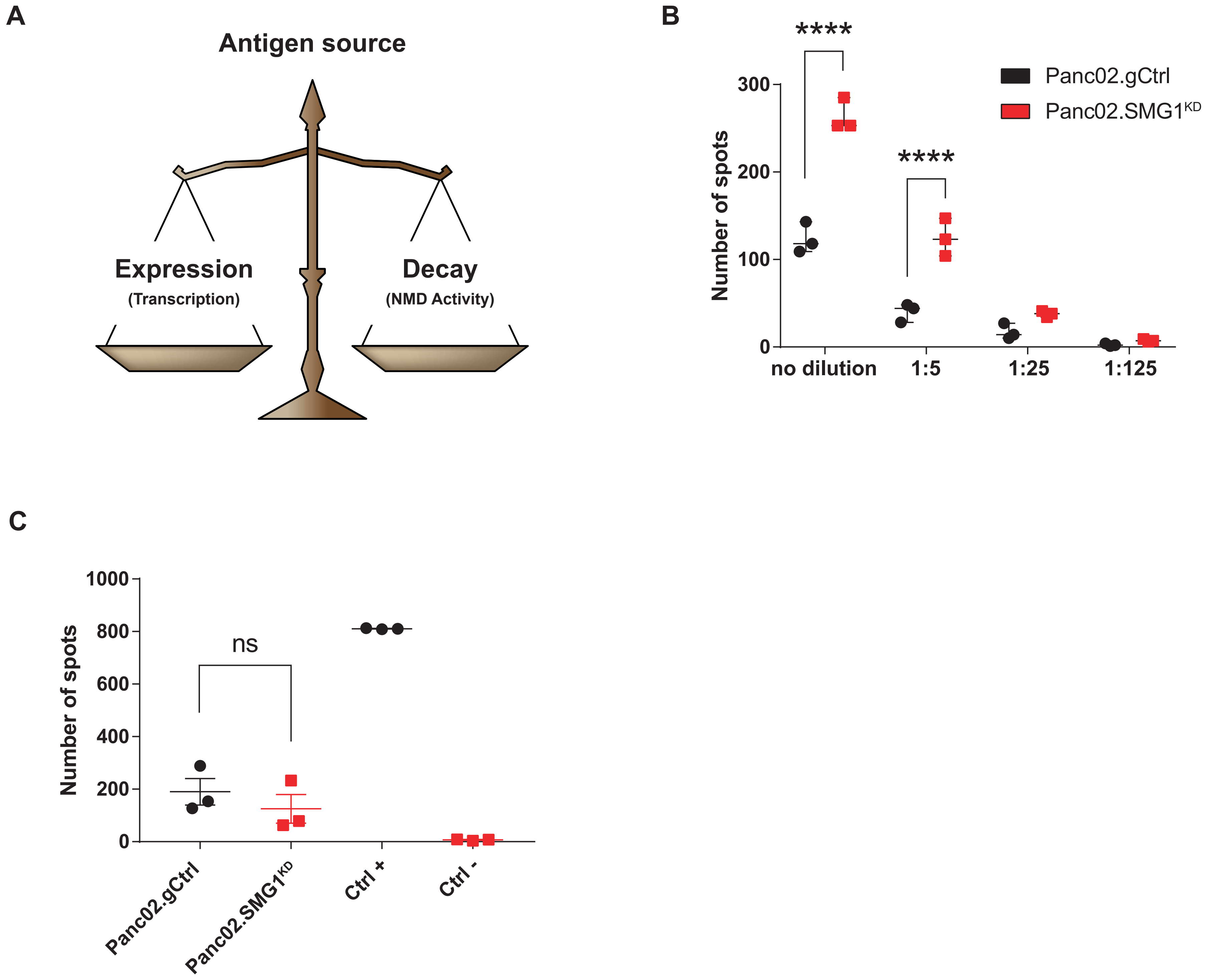

S7. NMD is not an absolute process leaking highly expressed PTC-antigens. Tools to study NMD regulation. (A) The fate of NMD substrate antigens is controlled by 2 main actors: increased transcriptional rate of mRNA, the greater amount of transcripts NMD has to degrade to silence mRNA expression; and NMD activity. Our work shows that NMD activity can be modulated: when it is upregulated, it can degrade transcripts in a more efficient way; on the other hand, when inhibited, it fails to control mRNA stability. This means that NMD might be saturated if enough transcript is generated and NMD cannot eliminate it fast enough to avoid its translation into a peptide. (B) Panc02.gCtrl and SMG1^{KD} where transfected with 2 plasmids: our luciferase-SIINFEKL reporter and a cargo plasmid. Total DNA remained unchanged (4 µg/well) but the NMD reporter was serially diluted (no dilution; 1:5; 1:25 and 1:125) in the cargo plasmid. Cell lines transfected this way were co-cultured with naïve OT-I splenocytes and IFN-γ production was measured by ELISPOT. (C) Basal levels of SIINFEKL presented by Panc02.gCtrl and SMG1^{KD} cells stably expressing the NMD reporter plasmid shown in (C). Panc02 were co-cultured with OT-I splenocytes and IFN-γ production was measured by ELISPOT.

2-way ANOVA corrected with Bonferroni's test was performed for tumor growth studies; 2-tail t-test for ELISPOT experiments. *$p < 0.05$, **$p < 0.01$, ***$p < 0.001$. ns = non-significant ($p > 0.05$).

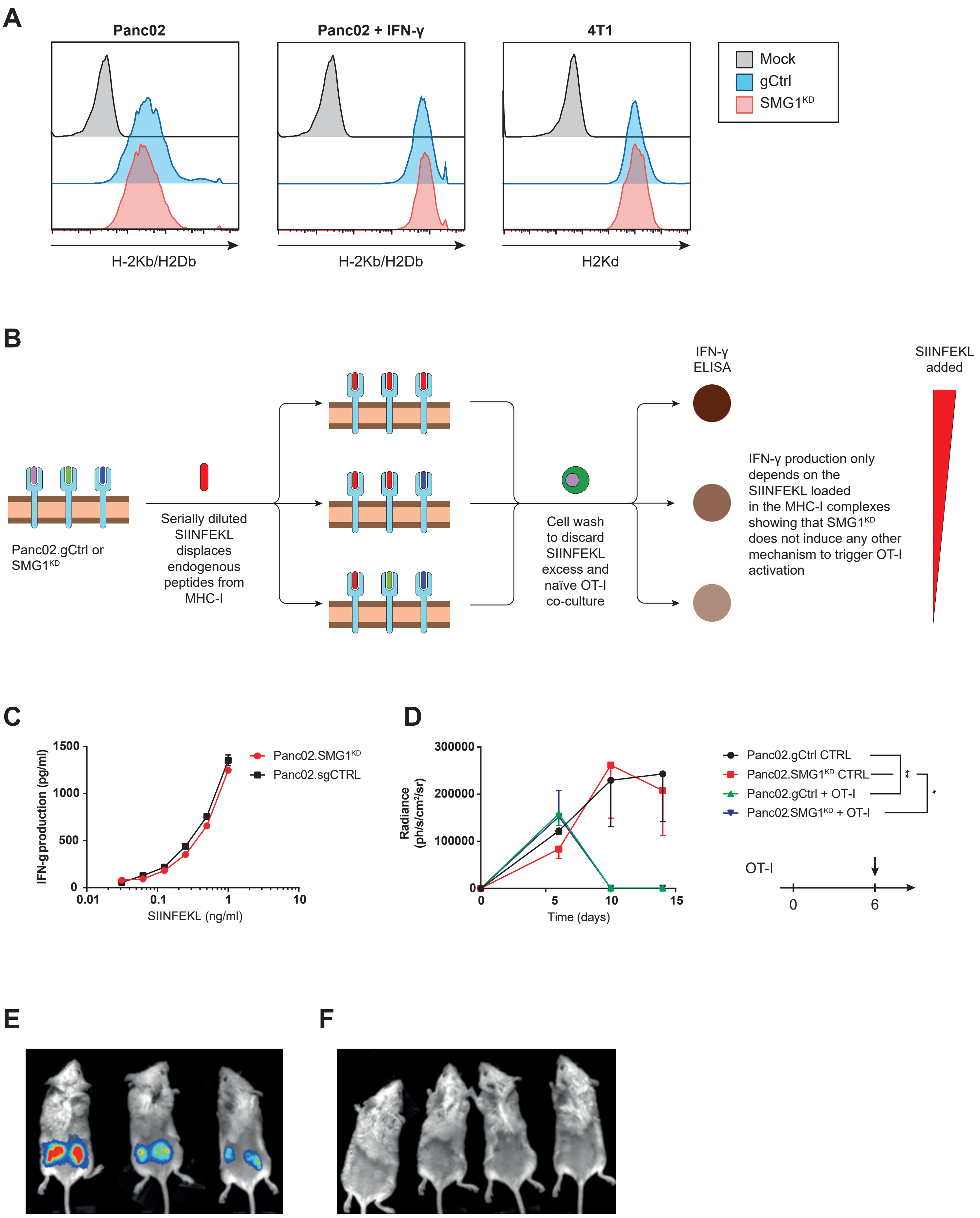

S8. SMG1 reduction does not alter antigen presentation of NMD-independent antigens. (A) MHC-I expression measured by flow cytometry in Panc02 gCtrl or SMG1^{KD} cells untreated or IFN-γ stimulated. 4T1 gCtrl or SMG1^{KD} cells MHC-I expression (right). (B) Experiment procedure for (C). Panc02.gCtrl and SMG1^{KD} were SIINFEKL-coated in different dilutions. Cells were washed to eliminate SIINFEKL excess and co-cultured with naïve OT-I splenocytes. IFN-γ production was quantified by ELISA. (C) IFN-γ production by activated OT-I splenocytes measured by ELISA illustrated in (B). (D) Rag2/IL2rg$^{-/-}$ mice were injected with Panc02.gCtrl in their right flank and SMG1^{KD} in their left one expressing the mutant PTC-free luciferase-SIINFEKL reporter plasmid stably. OT-I adoptive cell transfer was carried out on day 6. Luciferase was measured over time. n = 3-4/group. (E) Caption showing luciferase levels of control group on day 10. (F) Caption showing luciferase levels OT-I-treated mice on day 10. 2-way ANOVA corrected with Bonferroni's test was performed in (D). *p < 0.05, **p < 0.01, ***p < 0.001. ns = non-significant (p > 0.05).

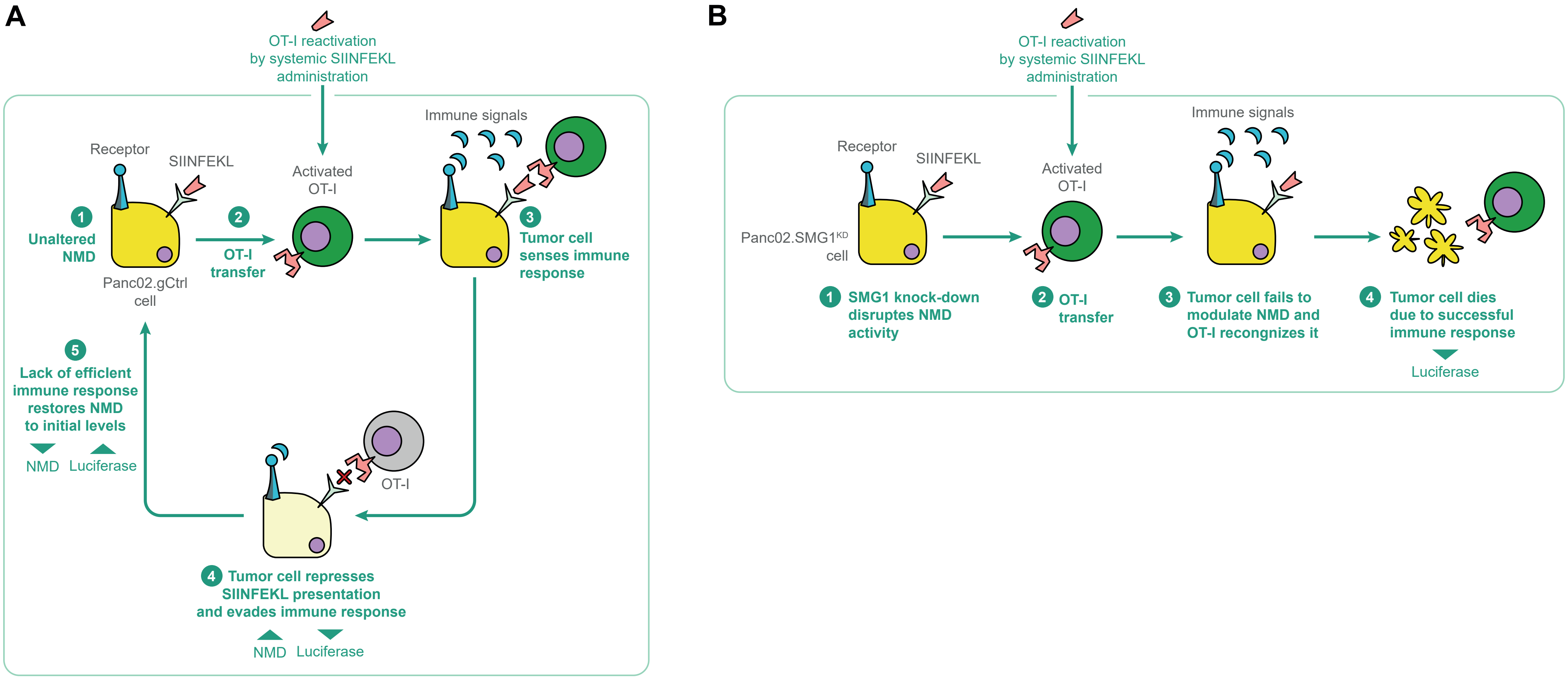

S9. Tumors trigger NMD upregulation as an immune escape mechanism to suppress PTC-containing neoantigen presentation.
(A) Proposed mechanism for Panc02.gCtrl expressing our luciferase-SIINFEKL reporter injected in Rag2/IL2rg$^{-/-}$ mice.
(B) Proposed mechanism for Panc02.SMG1^{KD} expressing our luciferase-SIINFEKL reporter injected in Rag2/IL2rg$^{-/-}$ mice.

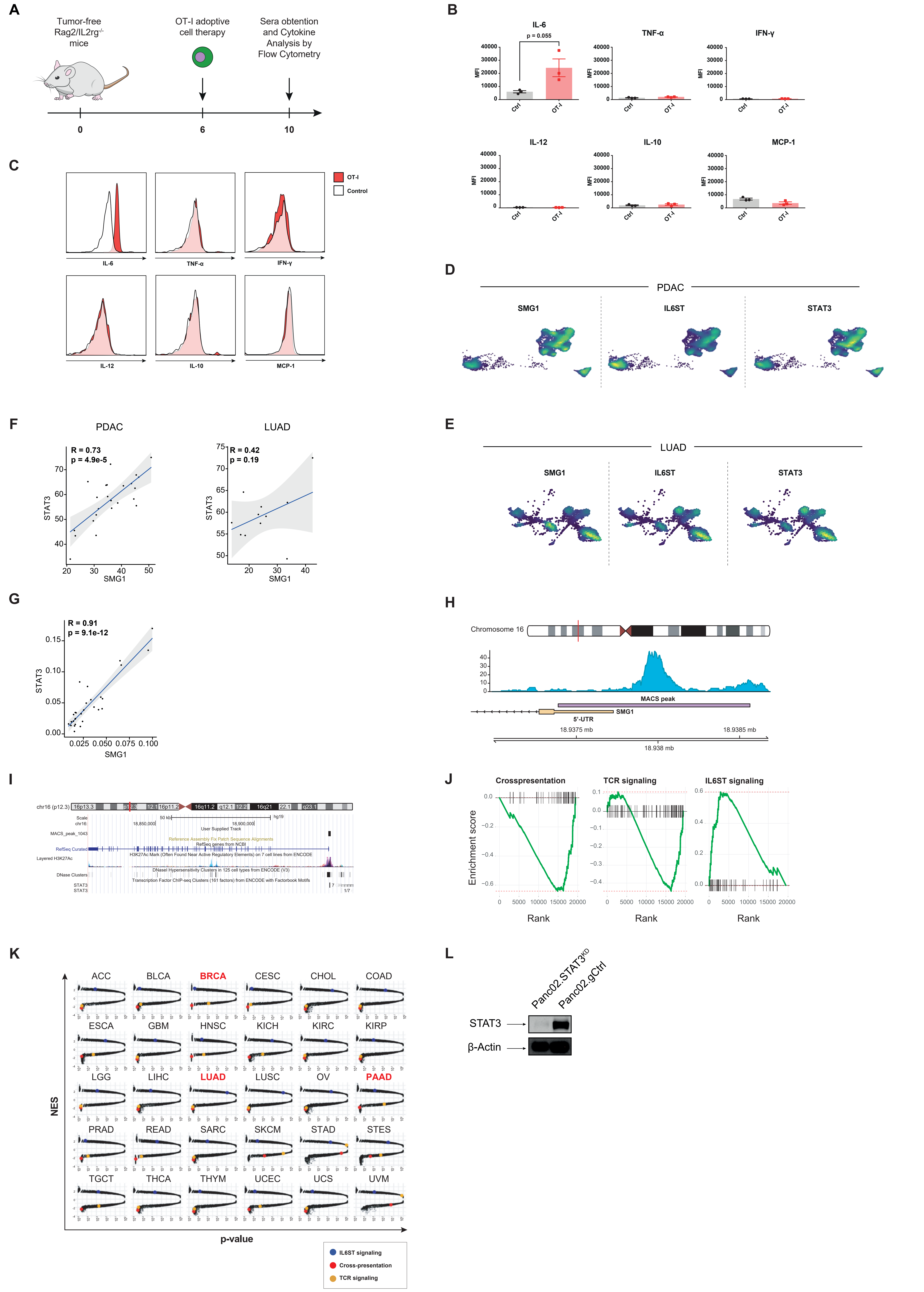

S10. NMD is upregulated by the IL-6/STAT3 axis. (A) Experiment schedule for (B). (B) Rag2/IL2rg-/- were injected with activated OT-I intravenously. On day 10 after the initiation of the experiment mice were bled and the presence of several cytokines was analyzed by flow cytometry. n = 3. (C) Representative individual from (B). (D) UMAP depicting PDAC patients scRNAseq from Peng et al., 2019, showing co-expression of SMG1 and IL6ST and STAT3. n = 11. (E) UMAP depicting LUAD patients scRNAseq from Kim et al., 2020 showing co-expression between the expression of SMG1 and IL6ST and STAT3 in tumor cells. n = 24. (F) PDAC and LUAD patients show a strong correlation in the expression of STAT3 and SMG1. scRNAseq data from Peng et al., 2019 and Kim et al., 2020, respectively. (G) STAT3 expression strongly correlates with SMG1 in a cohort of IL-6-expressing human tumor cell lines. mRNA levels measured by qRT-PCR. n = 29. (H) MACS peak data showing a STAT3 binding motive in SMG1 promoter. Data from Tripathi et al., 2017. (I) UCSC genome browser view of the STAT3 binding site in the promoter of SMG1. (J) GSEA, from left to right, showing cross-presentation, TCR signaling and IL6ST pathway genes correlation with SMG1. (K) Ranking of GSEAs in different cancer types from TCGA showing the position of IL6ST signaling, cross-presentation and TCR signaling based on their correlation with SMG1 expression. Data from TCGA. (L) WB showing STAT3 knockdown in Panc02 cells.

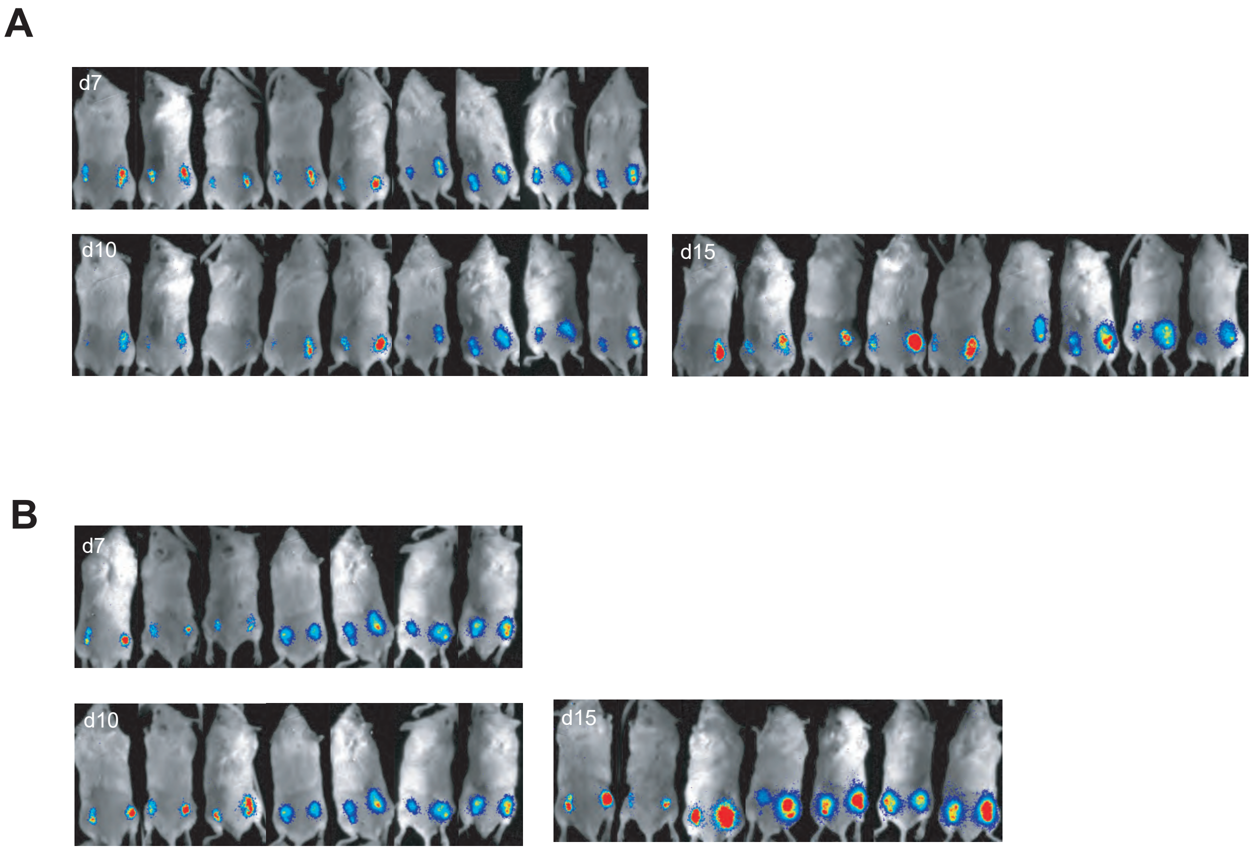

S11. STAT3[KD] or gCtrl Panc02 were injected in the left and right flanks, respectively, of Rag2/IL2rg$^{-/-}$ mice. (A) Activated OT-I or Pmel (B) splenocytes were administered intravenously as shown in (7J). Images of mice captured on days 7, 10 and 15 showing luciferase signal evolution in both tumors.
